# Brown midrib mutants in sorghum and their applications in renewable energy production

**DOI:** 10.3389/fpls.2026.1807795

**Published:** 2026-04-28

**Authors:** Akula Venkata Umakanth, Jinu Jacob, Passoupathy Rajendrakumar, Domathoti Balakrishna, Vikas Kumar, Bhavadharani Dhandapani, Kalesan Nikhita, Avinash Singode, Tara Satyavathi Chellapilla, Santosh Kumar Gupta

**Affiliations:** ICAR–Indian Institute of Millets Research, Hyderabad, India

**Keywords:** bioenergy, biomass recalcitrance, brown midrib mutants, fodder digestibility, genome editing, lignin biosynthesis, phenylpropanoid pathway, *Sorghum bicolor*

## Abstract

Lignin is a complicated phenolic polymer that is found in the secondary cell walls of plants. It is necessary for plant mechanical strength, vascular integrity, and stress tolerance. In sorghum (*Sorghum bicolor* L. Moench), the amount and composition of lignin greatly affect forage digestibility, biomass recalcitrance to bioenergy production and biochemical conversion process efficiency. Brown midrib (*bmr*) mutants, which exhibit reddish-brown pigmentation of the vasculature, have been a classic and potent tool for identifying lignin biosynthesis and engineering cell wall traits. Over the last 60 years, sorghum *bmr* mutants have been analyzed extensively and their characterization led to the identification of crucial genes associated with monolignol biosynthesis, such as *bmr*6 (cinnamyl alcohol dehydrogenase), *bmr*12 (caffeic acid O-methyltransferase), *bmr*2 (4-coumarate-CoA ligase), as well as having an indirect effect on lignification through one-carbon metabolism, i.e. *bmr*19. These changes modify lignin concentration, monomer composition, and interunit connections, leading to improved cell wall digestibility and decreased biomass recalcitrance. Lignin alteration involves physiological trade-offs that affect stem strength, lodging resistance, and disease susceptibility, and these are significantly influenced by genetic background and environmental factors. Recent advancements in genomics, systems biology, and genome-editing technologies have enabled precise manipulation of lignin pathways, uncovering compensatory metabolic networks that sustain plant fitness. This study consolidates existing knowledge on the molecular genetics, biochemical control, and physiological effects of brown midrib mutations in sorghum, focusing specifically on their implications in forage enhancement and renewable bioenergy generation. Through the amalgamation of traditional genetics and contemporary engineering methodologies, *bmr* sorghum serves as a paradigm C_4_ grass for sustainable lignocellulosic bioenergy and climate-resilient agriculture.

## 1 Introduction

Lignin is an aromatic, heterogeneous biopolymer that forms a significant component of secondary cell walls in vascular plants, playing a crucial role in upright development, effective water transport, and tolerance to biotic and abiotic stressors (Boerjan et al., 2003; Vanholme et al., 2019). Lignin, along with cellulose and hemicellulose, constitutes the structural foundation of lignocellulosic biomass. In grasses like sorghum (*Sorghum bicolor* L. Moench), lignin generally constitutes 15–25% of stem dry mass and is crucial in influencing cell wall rigidity, vascular efficiency, and overall plant structure (Scully et al., 2016). The chemical complexity that renders lignin essential for plant existence also imparts resistance to enzymatic hydrolysis, thereby restricting ruminant fiber digestibility and diminishing the efficiency of biomass conversion to liquid biofuels.

Sorghum is a significant C_4_ cereal crop recognized worldwide for its adaptability as a grain, fodder, and bioenergy feedstock. Sorghum’s elevated biomass productivity, resilience to drought and heat, and adaptability to suboptimal settings render it especially appealing for sustainable agriculture and renewable energy systems. Nonetheless, lignin-related recalcitrance remains a significant obstacle to enhancing forage quality and biochemical conversion efficiency. As a result, genetic approaches focused on altering lignin content and composition have garnered ongoing study attention.

Brown midrib (*bmr*) mutants provide a valuable genetic model for studying lignin modification in grasses. Originally identified in maize in 1924 with a distinctive reddish-brown coloration of leaf midribs, stems, and vascular tissues. It was first documented in maize (Jorgenson, 1931). These mutants were found to have brown pigmentation in the cob, roots, stem tassel, and midrib of the leaves. It was demonstrated that the *bmr* gene improved *in vitro* digestibility and effectively decreased the percentage of lignin (Lechtenberg et al., 1972). In Sorghum, *bmr* was introduced into several geographical settings (Porter et al., 1978; Bittinger et al., 1981). The newly developed sorghum genotypes were more digestible and contained less lignin compared to their parent genotypes (Fritz et al., 1981). The altered lignin deposition and the buildup of phenolic intermediates due to disruptions in monolignol production. Widely recognizable and closely linked to lignin chemistry, *bmr* mutants are extensively utilized to understand the genetic, biochemical, and physiological aspects of lignification (Saballos et al., 2008; Sattler et al., 2014). *bmr* Similar phenotypes have also been observed in other grasses, where the *bmr* trait correlates with changes in lignin biosynthesis and cell wall composition. This phenotype’s clear visual marker makes it a key tool for investigating the foundational processes of lignification.

*bmr* mutants were historically identified via spontaneous variation, chemical mutagenesis, or radiation-induced mutations, and were subsequently classified in the order of discovery (e.g., *bmr1*, *bmr2*, *bmr6*, *bmr12*). This phenotypic nomenclature predates molecular characterization and does not align with route position or functional significance. Subsequent allelism assays and gene cloning indicated that several *bmr* mutants are allelic variations of the same gene, differing in mutation severity and pleiotropic consequences. Consequently, contemporary research often uses a dual nomenclature that combines traditional *bmr* identifiers with gene-based annotations, exemplified by *bmr*6 (SbCAD2) and *bmr*12 (SbCOMT) (Bout and Vermerris, 2003).

Lignin biosynthesis at the biochemical level occurs via the phenylpropanoid pathway, initiating with phenylalanine ammonia-lyase and concluding with the synthesis of the three principal monolignols: p-coumaryl (H), coniferyl (G), and sinapyl (S) alcohols, which are subsequently polymerized in the cell wall by peroxidases and laccases (Zhao, 2016). Lignification in sorghum is tightly regulated at the tissue and developmental levels and is closely associated with hydroxycinnamate cross-linking, a feature unique to grass cell walls that contributes to biomass recalcitrance. Lignin, a multi-functional biopolymer, provides mechanical stability, maintains vascular integrity as well as mediating plant defense and stress adaptation. Liu et al. (2018) recently reviewed that lignin composition, as well as spatial deposition, is tightly controlled and context-dependent, and impairment in lignin biosynthetic pathways could influence growth, development, and stress responses. These data highlight the necessity of harmonizing cell wall engineering with the general fitness of plants in lignin-altered crops like brown midrib sorghum.

Over the last twenty years, molecular analysis of sorghum *bmr* mutants has revealed various genetic pathways that alter lignin content and composition, including direct inhibition of monolignol biosynthetic enzymes and indirect effects through one-carbon metabolism and pathway interactions. These findings have not only enhanced the fundamental understanding of lignin biology but also facilitated the development of sorghum genotypes with superior fodder digestibility and greater suitability for bioethanol and advanced biofuel production (Widodo et al., 2025).

While several reviews have discussed lignin biosynthesis and engineering in grasses, this review offers a focused, integrated perspective on brown midrib (*bmr*) sorghum as a model for lignin modification in C_4_ crops. It (i) summarizes recent advances in the molecular genetics of key *bmr* loci and their biochemical effects in sorghum; (ii) highlights emerging insights connecting lignin biosynthesis with related metabolic processes like one-carbon metabolism and flavonoid-derived lignin monomers; (iii) combines systems biology and multi-omics research that uncover regulatory networks governing lignification; and (iv) explores the practical importance of *bmr* sorghum for forage enhancement, lignocellulosic bioenergy, and lignin valorization in integrated biorefineries. Additionally, we review how recent genome-editing techniques offer new opportunities for precise lignin engineering and crop improvement.

This review rigorously analyzes the existing understanding of brown midrib mutants in sorghum, emphasizing their molecular genetics, biochemical regulation of lignin biosynthesis, physiological trade-offs, and practical implications in renewable energy generation. By merging traditional genetics with contemporary systems biology and genome-editing techniques, we underscore the promise of *bmr* sorghum as a model system for lignin research in C_4_ grasses and a fundamental element of sustainable bioenergy agriculture.

This review is structured into various thematic sections to clarify lignin modification in sorghum. Initially, it covers the biochemical basis of lignin biosynthesis in sorghum and emphasizes specific features of grass cell walls. Next, it explores the molecular genetics of brown midrib (*bmr*) mutants and how these mutations alter lignin composition and deposition. Following that, the discussion moves to the regulation of lignification and the physiological and agronomic trade-offs linked to *bmr* mutations. Lastly, the review considers the impacts of lignin modification on forage quality, lignocellulosic bioenergy, and innovative genome-editing techniques for targeted lignin engineering in sorghum.

## 2 Lignin biosynthesis and molecular basis of bmr mutants

### 2.1 Lignin biosynthesis pathway and grass specific characteristics

Lignin is a complex and heterogeneous phenolic polymer that, alongside cellulose and hemicellulose, constitutes the structural basis of secondary cell walls in vascular plants. Lignin deposition in sorghum is particularly high in xylem vessels and sclerenchyma tissues, providing mechanical support, preventing collapse of vessels under tension (negative pressures), and limiting the effects of biotic and abiotic stress (Boerjan et al., 2003).

Lignin is the primary biochemical barrier to cell walls disassembly for biomass utilization. Aromatic features and a higher level of cross-linking, combined with the high content of polysaccharides, restrict enzyme access to cellulose and hemicellulose, thereby reducing digestibility and requiring more severe pretreatment for bioenergy applications (Chen and Dixon, 2007; Vanholme et al., 2019). Thus, understanding the biosynthetic and regulatory framework of lignin in sorghum is essential for improving forage quality and renewable energy production. Understanding the biochemical architecture of lignin biosynthesis provides the foundation for interpreting how genetic mutations modify cell wall properties. In sorghum, brown midrib mutants have served as powerful genetic tools to dissect this pathway and identify key regulatory nodes controlling lignin composition.

### 2.2 Phenylpropanoid pathway and monolignol biosynthesis

Lignin biosynthesis is derived from the phenylpropanoid pathway, which channels carbon from primary metabolism into secondary metabolites, including lignin, flavonoids, and hydroxycinnamic acids. The route commences with phenylalanine ammonia-lyase (PAL), which facilitates the deamination of phenylalanine to cinnamic acid. This stage marks a critical metabolic junction and is meticulously regulated in response to developmental signals and environmental factors.

The phenylpropanoid pathway serves as a pivotal metabolic center that provides precursors for lignin and many specialized phenolics. Vogt (2010) delivered a thorough synthesis of phenylpropanoid biosynthesis, highlighting its wide pathway branching, regulatory intricacies, and metabolic versatility, which allow plants to equilibrate structural, defense, and signaling roles. This framework is especially pertinent for analyzing brown midrib (*bmr*) sorghum phenotypes, where disruptions at enzymatic stages frequently induce compensatory shifts in metabolic flux between lignin and flavonoid pathways, ultimately influencing lignin composition and cell wall characteristics.

Recent findings have challenged the established concept of lignin production by uncovering additional enzymatic steps in the monolignol pathway. Vanholme et al. (2013) established that caffeoyl shikimate esterase (CSE) functions as a genuine enzyme, catalyzing the conversion of caffeoyl shikimate to caffeic acid. This discovery revealed an alternate pathway in the phenylpropanoid biosynthesis and emphasized the inherent flexibility of lignin production. The original characterization of CSE in Arabidopsis has transformed pathway models in grasses and offers crucial insights into metabolic compensation and flux redirection in lignin-modified systems, such as brown midrib sorghum (Vanholme et al., 2013).

Subsequent to PAL-mediated pathway entry, cinnamic acid undergoes hydroxylation by cinnamate 4-hydroxylase (C4H) to yield p-coumaric acid, which is then converted to p-coumaroyl-CoA by 4-coumarate-CoA ligase (4CL). The initial enzymatic processes significantly regulate carbon flow into lignin production and are hence crucial factors in total lignin build-up (Zhang et al., 2020; Zhang et al., 2021). In sorghum, the inhibition of 4CL activity, as seen in *bmr*2 mutants, diminishes carbon flux into the monolignol pathway, leading to reduced lignin accumulation and broader modifications in phenylpropanoid-derived metabolites (Saballos et al., 2008). Information regarding brown midrib (*bmr*) loci related to enzymes in the sorghum lignin biosynthesis pathway as indicated in **Table 1**.

**Table 1:** Mapping of brown midrib (*bmr*) loci to enzymes in the sorghum lignin biosynthetic pathway.

Enzyme (pathway step)	Representative sorghum gene (Sobic.)	*bmr* locus mapped?	Evidence/notes
PAL – Phenylalanine ammonia-lyase	Sobic.004G220300	No	PAL gene family characterized; no *bmr* locus mapped
C4H – Cinnamate-4-hydroxylase (CYP73A)	Sobic.002G126600	No	Expressed in lignifying tissues; no *bmr* assignment
4CL – 4-Coumarate: CoA ligase	Sobic.004G062500	Yes – *bmr*2	*bmr*2 cloned; missense mutations disrupt 4CL activity
HCT – Hydroxycinnamoyl-CoA transferase	Sobic.004G212300	No	Identified but not linked to *bmr* phenotype
C3H – p-Coumarate 3-hydroxylase	Sobic.009G181800	No	No confirmed *bmr* mapping
CCR – Cinnamoyl-CoA reductase	Sobic.007G141200	No	Candidate genes reported; no cloned *bmr* locus
COMT – Caffeic acid O-methyltransferase	Sobic.007G047300	Yes – *bmr*12	Multiple alleles cloned; reduced S-lignin
F5H – Ferulate 5-hydroxylase	Sobic.001G196300	No	Alters S/G ratio when overexpressed; no *bmr* locus
CAD – Cinnamyl alcohol dehydrogenase	Sobic.004G071000	Yes – *bmr*6	SbCAD2 cloned; aldehydic lignin accumulation
CHI – Chalcone isomerase	Sobic.001G035600	Yes – *bmr*30	Alters tricin–lignin coupling

Subsequent to 4CL-mediated activation, monolignol biosynthesis advances through a succession of hydroxylation, methylation, and reduction events that produce the three principal lignin monomers: p-coumaryl alcohol (H unit), coniferyl alcohol (G unit), and sinapyl alcohol (S unit). Prominent enzymes implicated in these transformations encompass hydroxycinnamoyl-CoA shikimate/quinate hydroxycinnamoyl transferase (HCT), p-coumarate 3-hydroxylase (C3H), caffeoyl shikimate esterase (CSE), caffeoyl-CoA O-methyltransferase (CCoAOMT), ferulate 5-hydroxylase (F5H), cinnamoyl-CoA reductase (CCR), and cinnamyl alcohol dehydrogenase (CAD) (Boerjan et al., 2003). Among these enzymes, COMT and CAD play essential roles in shaping lignin composition and structure. COMT is essential for the methylation processes involved in syringyl (S) lignin production, while CAD facilitates the final reduction of hydroxycinnamaldehydes to their respective alcohols before polymerization. Mutations at these stages, including *bmr*12 (COMT) and *bmr*6 (CAD2), lead to significant alterations in lignin monomer composition, the inclusion of unconventional units, and diminished polymer integrity (Bout and Vermerris, 2003; Palmer et al., 2008; Saballos et al., 2008).

### 2.3 Polymerization and lignin deposition

Monolignols produced in the cytoplasm are conveyed to the apoplast, where they undergo oxidative polymerization facilitated by class III peroxidases and multicopper oxidases (laccases). This radical-mediated, non-template-driven process yields a structurally heterogeneous lignin polymer characterized by various interunit connections, primarily *β*-O-4, in addition to *β*-*β* and *β*-5 bonds.

Lignification in sorghum and other grasses is closely regulated alongside secondary cell wall development and is confined to cell types. Lignin deposition is not a passive chemical event; it is a meticulously managed cellular process that entails fine spatial and temporal control over monolignol production, transport, and oxidative coupling within the cell wall matrix. Barros et al. (2015) shown that lignin polymerization is intricately associated with cell wall structure, vesicular transport, and the localized function of oxidative enzymes, notably laccases and peroxidases, which regulate polymer formation at specific wall regions. This control affects lignin structure, cross-linking patterns, and its association with other wall polymers.

The degree and distribution of lignin deposition vary with developmental stage, tissue type, and environmental factors. Lignin in grasses is deposited in close proximity to hemicelluloses, particularly arabinoxylans, forming intricate lignin–carbohydrate networks that increase secondary wall rigidity and contribute to biomass recalcitrance (Kirui et al., 2022).

The cell walls of grass are characterized by a significant presence of hydroxycinnamic acids, notably ferulic acid and p-coumaric acid. Hydroxycinnamates are pivotal in grass lignification by facilitating the cross-linking of lignin and cell wall polysaccharides. Ralph (2010) established that ferulates and p-coumarates are essential for the onset of lignin polymerization, interunit coupling, and the development of lignin–carbohydrate complexes in grasses. Their insertion increases cell wall rigidity and biomass recalcitrance, although these chemically labile connections also present prospective targets for lignin modification techniques, as seen in brown midrib (*bmr*) sorghum.

Ferulates esterified to arabinoxylans participate in oxidative coupling, forming cross-links between polysaccharide chains and between polysaccharides and lignin, thereby strengthening the secondary cell wall matrix (Hatfield and Fukushima, 2005). Ferulate-mediated networks significantly influence the remarkable recalcitrance of grain residues, such as sorghum stover.

In addition to hydroxycinnamates, tricin has become a significant grass-specific lignin monomer that affects polymer initiation, structure, and reactivity. Tricin is a flavonoid-derived molecule that integrates into lignin and acts as a nucleation site for polymerization. Eudes et al. (2017) established that SbCOMT (*bmr*12) directly influences tricin production and its integration into sorghum lignin, hence connecting flavonoid metabolism with lignification. The loss of *bmr*12 function led to diminished tricin–lignin synthesis and modified lignin composition, offering mechanistic evidence that *bmr*12 mutations alter lignin architecture by affecting syringyl unit content and changing grass-specific lignin characteristics.

Additional genetic support for cross-pathway regulation of lignification in sorghum is provided by the identification of *bmr*30 as a chalcone isomerase, a crucial enzyme in flavonoid biosynthesis (Tetreault et al., 2021). This discovery indicated that disruptions in flavonoid metabolism might indirectly modify lignin deposition and composition, highlighting the significance of synchronized regulation between phenylpropanoid and flavonoid pathways in influencing lignin structure and biomass recalcitrance in grasses.

The biosynthesis of lignin in sorghum is governed by a complex regulatory network that integrates transcriptional regulation with metabolic flux control (Zhong and Ye, 2009; Vanholme et al., 2019). NAC domain transcription factors serve as primary regulators of secondary cell wall development at the transcriptional level, initiating downstream MYB transcription factors that directly govern lignin biosynthetic genes. This hierarchical network guarantees accurate spatial and temporal regulation of lignification throughout vascular development (Zhong et al., 2010; Nakano et al., 2015; Xiao et al., 2021).

Lignin production is intricately connected to primary metabolism, in addition to transcriptional regulation. The synthesis of monolignols necessitates significant reducing power and methyl group donors (S-adenosylmethionine, SAM), hence linking lignin biosynthesis to central carbon metabolism, the pentose phosphate pathway, and one-carbon metabolism. Disruptions in these interrelated processes, as seen in *bmr*19 mutants, might indirectly modify lignin concentration and composition by limiting methylation capacity and redox equilibrium (Adeyanju et al., 2021).

The biochemical and regulatory characteristics of lignin biosynthesis in sorghum provide a mechanistic foundation for understanding the implications of brown midrib mutations. Partial disruption of particular enzymatic processes or regulatory points might significantly diminish biomass recalcitrance without entirely eliminating lignification. *bmr* mutants demonstrate that targeted alteration of lignin amount and quality is sufficient to improve digestibility and saccharification efficiency, whereas total lignin removal is neither essential nor advantageous given its critical physiological roles (Sattler et al., 2014; Saballos et al., 2008; Dien et al., 2009; Godin et al., 2016; Li and Huang, 2017).

**Figure 1:**
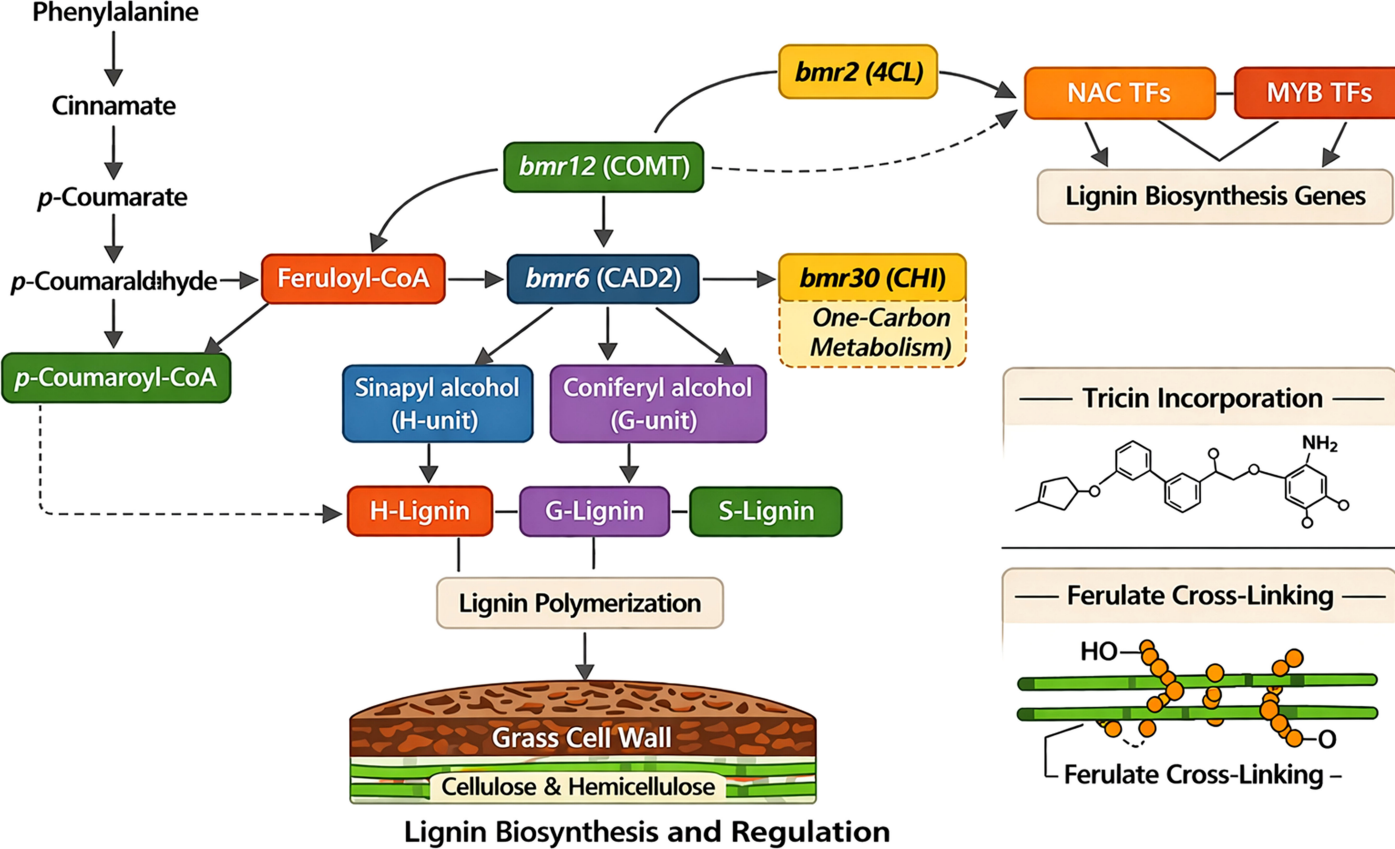
Overview of lignin biosynthesis and regulatory mechanisms controlling lignin deposition in sorghum. This figure depicts the phenylpropanoid pathway responsible for lignin biosynthesis in sorghum. It starts with phenylalanine and proceeds through several enzymatic reactions, leading to the production of the three main monolignols: p-coumaryl alcohol (H), coniferyl alcohol (G), and sinapyl alcohol (S). Key enzymes involved include PAL, C4H, 4CL, HCT, C3H, CCR, CAD, COMT, and F5H, which are highlighted in the diagram. It also shows transcriptional regulation by NAC and MYB transcription factors and post-transcriptional control via miRNA-mediated regulation of laccase genes involved in lignin polymerization. These regulatory layers coordinate lignin biosynthesis, affecting cell wall structure, plant growth, and biomass use. The enzymatic steps converting phenylalanine into lignin polymers in the secondary cell wall involve monolignol synthesis through the phenylpropanoid pathway, emphasizing enzymes such as PAL, C4H, 4CL, HCT, C3H, CCR, CAD, COMT, and F5H. Lignin gene expression is generally hierarchically controlled by NAC and MYB transcription factors. Post-transcriptionally, miR397 represses laccase genes, influencing lignin polymerization. Variations in these regulatory networks can alter lignin amount and composition, impacting cell wall structure, stress responses, and postharvest qualities.

Insights into the lignin biosynthesis process and its regulation in sorghum collectively provide a conceptual framework for understanding *bmr* phenotypes and for formulating strategic approaches to enhance lignin characteristics for forage and renewable energy applications (**Figure 1**).

### 2.4 Molecular genetics of brown midrib mutants

Brown midrib (*bmr*) mutants in sorghum were first recognized via spontaneous variation and induced mutagenesis initiatives designed to enhance fodder digestion. The distinctive reddish-brown coloration of leaf midribs, stems, and vascular tissues offered a visually assessable phenotype linked to modified lignin deposition, enabling swift selection in breeding populations. Early genetic analyses demonstrated that *bmr* phenotypes are typically inherited as recessive traits controlled by single nuclear loci, although phenotypic expressivity varies with genetic background and environmental conditions.

Comparative investigations identified *bmr* mutations as a conserved mechanism for altering lignocellulosic cell walls in C_4_ grasses. Sattler et al. (2010b) conducted an extensive investigation of *bmr* mutants in maize, sorghum, and pearl millet, illustrating that reduced lignin content and modified lignin composition regularly improve cell wall digestibility and processing efficiency. Their research highlighted that agronomic performance is contingent upon the individual gene affected and the genetic background, emphasizing the necessity for balanced breeding techniques. This study positioned *bmr* sorghum as a model system for lignin modification with broad relevance to livestock nutrition and bioenergy applications (Sattler et al., 2010a).

The disruption of lignin production in *bmr* mutants may also affect plant defense mechanisms. Modifications in phenylpropanoid metabolism affect cell wall integrity and soluble phenolic profiles, impacting plant–pathogen interactions. Funnell-Harris et al. (2017) revealed that *bmr* sorghum lines display varied reactions to stalk-rotting pathogens in contrast to near-isogenic normal lines, partially attributable to alterations in soluble phenolic chemicals resulting from lignin pathway disruption. These findings underscore the trade-offs between enhanced digestibility and disease resistance in lignin-altered sorghum.

At the molecular level, the interruption of phenylpropanoid pathway enzymes often elicits compensatory metabolic responses owing to significant route branching and control (Vogt, 2010). The discovery of alternative enzymatic processes, including caffeoyl shikimate esterase (CSE), exemplifies the adaptability of lignin production and elucidates metabolic compensation after route disruption (Vanholme et al., 2013). Bonawitz and Chapple (2010) revealed that mutations in monolignol biosynthesis genes frequently produce pleiotropic effects, correlating modified lignin composition with developmental and mechanical traits. Collectively, this research formulated a conceptual framework linking genotype, pathway disruption, and phenotypic results in lignin-altered plants.

Before molecular characterization, *bmr* mutants were assigned numerical identities (e.g., *bmr*1, *bmr*2, *bmr*6, *bmr*12) according to their order of discovery rather than their biochemical function. This terminology, while historically beneficial, concealed the functional links among mutants and allelic variation. Recent progress in molecular genetics and comparative genomics facilitated the cloning and functional annotation of several *bmr* loci, demonstrating that many correspond to essential monolignol biosynthesis enzymes or regulators that indirectly affect lignification. Numerous brown midrib loci have been molecularly cloned and functionally described in sorghum, elucidating both direct and indirect pathways to lignin alteration (**Table 2**).

**Table 2:** Molecularly characterized brown midrib (*bmr*) mutants in *Sorghum bicolor*.

Gene	Allelic group/status	Enzyme/pathway step	Gene ID (sobic)	Molecular status	Key effect on lignin	Phenotypic/applied significance	Key References
*bmr*2	Multiple alleles	4-Coumarate: CoA ligase (4 CL;Phenylpropanoid entry step)	*Sobic.004G062500*	Cloned, validated	Reduced total lignin; altered phenylpropanoid flux	Reduced stem rigidity; improved digestibility	Saballos et al., 2012
*bmr*6	≥8 alleles	Cinnamyl alcohol dehydrogenase 2 (CAD2; Final monolignol reduction)	*Sobic.004G071000*	Cloned, validated	Reduced lignin; Increased aldehydic residues	Softer stems; high saccharification efficiency	Bout and Vermerris, 2003; Saballos et al., 2008
*bmr*12	Multiple EMS alleles	Caffeic acid O-methyltransferase (COMT; S-lignin biosynthesis)	*Sobic.007G047300*	Cloned, validated	Reduced S-lignin; Decreased S/G ratio	Widely used in forage and bioenergy breeding	Palmer et al., 2008; Saballos et al., 2012
*bmr*19	Allelic group	Folylpolyglutamate synthase (FPGS;One-Carbon Metabolism; Indirect lignin regulation)	*Sobic.001G535500* (candidate)	Candidate gene	Altered lignification (indirect effect)	Suggests regulatory control of lignin metabolism	Adeyanju et al., 2021
*bmr*30	Two alleles	Chalcone isomerase (CHI;tricin-lignin coupling)	*Sobic.001G035600*	Cloned, functionally validated	Modified lignin polymerization	Links flavonoid metabolism to lignification	Tetreault et al., 2021
*bmr*1-19	Multiple groups	–	–	Phenotype-based	Variable	Foundational allelism resource	Porter et al., 1978
*bmr*29-32	Tentative groups	–	–	Candidate loci	Unknown	Mapping-based evidence	Sattler et al., 2014
*bmr*33-38	Not established	–	–	EMS/TILLING lines	Unknown	Mutant resource collections	Xin et al., 2009

#### 2.4.1 bmr6: disruption of cinnamyl alcohol dehydrogenase

The *bmr*6 locus is one of the most thoroughly known brown midrib alterations in sorghum and was among the first to be molecularly elucidated. *bmr*6 encodes SbCAD2, a cinnamyl alcohol dehydrogenase that facilitates the final reduction of hydroxycinnamaldehydes to their respective monolignols before lignin polymerization (Bout and Vermerris, 2003). Loss-of-function or diminished-function mutations in SbCAD2 result in the buildup of cinnamaldehydes, which are integrated into the lignin polymer as atypical units.

*bmr*6 mutants display reduced total lignin concentration and changed lignin composition, marked by elevated aldehyde residues and altered interunit connections (Palmer et al., 2008). The structural alterations diminish the lignin–carbohydrate matrix, leading to enhanced enzymatic saccharification and elevated *in vitro* dry matter digestibility. *Bmr*6 mutants typically exhibit diminished stem strength and heightened vulnerability to lodging, however these effects are significantly affected by genetic background and can be substantially alleviated through selective breeding (Saballos et al., 2008; Sattler et al., 2010a).

#### 2.4.2 bmr12: impairment of caffeic acid O-methyltransferase

The *bmr*12 locus encodes SbCOMT, an O-methyltransferase essential for the production of sinapyl alcohol and, subsequently, syringyl (S) lignin units. Mutations in SbCOMT decrease S-lignin content and modify the S:G ratio, resulting in lignin polymers that are less condensed and more susceptible to chemical and enzymatic degradation (Bout and Vermerris, 2003; Saballos et al., 2008).

In comparison to *bmr*6, *bmr*12 mutants often maintain a relatively normal plant size and vascular development, while demonstrating significant enhancements in fodder digestibility and bioethanol output. The equilibrium between diminished recalcitrance and maintained agronomic efficacy has rendered *bmr*12 especially appealing for practical breeding and bioenergy purposes (Dien et al., 2009; Scully et al., 2016). Nonetheless, *bmr*12 lines may exhibit heightened susceptibility to specific infections, indicating the protective function of syringyl-rich lignin disease resistance.

#### 2.4.3 *bmr*2: modification of 4-coumarate-CoA ligase (4CL)

The *bmr*2 mutation influences 4-coumarate-CoA ligase (4CL), an enzyme located at a pivotal regulatory juncture that directs phenylpropanoid precursors into monolignol biosynthesis. Disruption of 4CL, which functions upstream of many branching pathways, results in extensive metabolic repercussions that extend beyond mere lignin reduction (Saballos et al., 2008).

*bmr*2 mutants generally display a significant reduction in total lignin content, alongside the increase of soluble phenolics and modified hydroxycinnamic acid profiles. Although these modifications improve digestibility and saccharification efficiency, *bmr*2 mutants frequently exhibit more pronounced pleiotropic effects than *bmr*6 or *bmr*12, such as diminished vitality and impaired vascular integrity. Consequently, *bmr*2 has had little implementation in commercial breeding efforts, it continues to be crucial in clarifying flux regulation within the phenylpropanoid pathway.

#### 2.4.4 bmr19: indirect modulation of lignification through one-carbon metabolism

In contrast to classical *bmr* mutations that directly impair monolignol biosynthesis enzymes, *bmr*19 exemplifies an indirect regulatory pathway for lignin modification. The *bmr*19 locus encodes folylpolyglutamate synthase, an essential enzyme in one-carbon metabolism necessary for the production of S-adenosylmethionine (SAM), the universal methyl donor for lignin O-methylation processes (Adeyanju et al., 2021).

The disruption of one-carbon metabolism in *bmr*19 mutants diminishes cellular methylation capacity, consequently limiting COMT- and CCoAOMT-mediated events in the monolignol pathway without directly inactivating lignin biosynthesis enzymes (Vanholme et al., 2012). *Bmr*19 mutants display modified lignin composition and slight decreases in overall lignin content, resulting in enhanced cell wall digestibility and relatively minor developmental drawbacks. These attributes highlight the possibility of *bmr*19 as a breeding target for optimizing lignin properties. Recent metabolic engineering research reinforces the significance of one-carbon metabolism as a regulatory hub in lignification. Tian et al. (2024) revealed that tailored decreases in SAM availability alter lignin content and syringyl/guaiacyl (S/G) ratios by restricting methylation processes in the monolignol biosynthesis pathway. The resultant lignin displayed modified structure and improved enzymatic digestibility, offering mechanistic understanding of the *bmr*19 phenotype and underscoring the potential of addressing methyl-donor pathways to diminish biomass recalcitrance in bioenergy sorghum.

### 2.5 Emerging bmr loci and genetic variability

Alongside the recognized *bmr*2, *bmr*6, *bmr*12, and *bmr*19 sites, various more *bmr* mutations have been documented in sorghum, although their molecular identities are not as firmly delineated. The genes *bmr*1, *bmr*3, and *bmr*5 are linked to modified lignin phenotypes but lack conclusive gene-level annotation. The ongoing utilization of high-resolution mapping, transcriptomics, and genome sequencing is anticipated to elucidate these loci and augment the array of lignin-modifying genes in sorghum. The finding of *bmr*30, which encodes chalcone isomerase, expanded the conceptual framework of *bmr* genetics by connecting flavonoid production to the start of lignin polymer through tricin incorporation (Tetreault et al., 2021). This discovery underscores that lignification is not a singular pathway but a metabolically interconnected process affected by various branches of secondary metabolism.

A common challenge in *bmr* investigations is the significant influence of allelic variation and genetic background on phenotypic outcomes. Different alleles at the same *bmr* locus can cause various levels of lignin alteration and agronomic effects, ranging from minor biochemical changes to major developmental abnormalities (Sattler et al., 2014). Additionally, the expression of *bmr* phenotypes is affected by environmental factors such as temperature, water availability, and nutrient levels, demonstrating the adaptive plasticity of lignin biosynthesis. These complexities emphasize the need to combine molecular genetics with quantitative trait analysis and breeding approaches to harness the benefits of *bmr* mutations while minimizing undesirable trade-offs. While individual *bmr* loci reveal how specific enzymes influence lignin biosynthesis, lignification in plants is orchestrated by intricate regulatory networks that coordinate transcriptional processes, metabolic flux, and cell wall formation (Funnell-Harris et al., 2010). Understanding these regulatory layers is crucial for interpreting the broader physiological impacts of lignin modification.

Collectively, research on *bmr* mutants has provided fundamental insights into the genetic architecture of lignin biosynthesis in grasses. By identifying key enzymatic and regulatory steps controlling lignin deposition, these studies have laid the foundation for modern lignin engineering approaches aimed at improving biomass utilization. The continued integration of classical genetics with genomics, systems biology, and genome-editing technologies is expected to accelerate the development of sorghum cultivars with optimized lignin characteristics for both forage production and renewable bioenergy applications.

## 3 Regulation, cell wall architecture, and agronomic implications

Lignification in sorghum is regulated by a complex architecture that encompasses transcriptional control, post-transcriptional regulation, enzyme activity, and metabolic flux. The lignin pathway functions within a comprehensive secondary metabolic network, responding dynamically to developmental signals and environmental stimuli, rather than as a linear biosynthetic route (Vanholme et al., 2019). As a result, genetic alterations caused by *bmr* mutations frequently elicit compensatory regulatory mechanisms that modify cell wall structure without eliminating lignification.

Lignin biosynthesis genes are co-regulated at the transcriptional level within the secondary cell wall (SCW) pathway. This program orchestrates the deposition of cellulose, hemicellulose, and lignin, guaranteeing mechanical integrity and vascular functioning. The disruption of certain enzymes in the monolignol pathway influences both lignin quantity and composition, as well as the expression of other cell wall-related genes, highlighting the interconnectedness of secondary cell wall production (Zhong and Ye, 2015).

### 3.1 Regulatory networks of transcription factors regulating lignification

Hierarchical transcription factor networks are pivotal in regulating lignin production in sorghum. NAC domain transcription factors serve as principal regulators of secondary cell wall development by activating a series of downstream MYB transcription factors, which then directly regulate structural genes implicated in the manufacture of lignin, cellulose, and hemicellulose (Zhong et al., 2010).

In sorghum, orthologs of well-characterized NAC regulators, including SND1, NST1, and VND proteins, have been demonstrated to regulate vascular development and lignification. MYB transcription factors, regulated by NACs, display both activating and repressive functions, meticulously modulating lignin concentration, and composition in a tissue-specific context. Significantly, investigations of *bmr* mutants reveal that diminished lignin accumulation is not solely due to decreased enzymatic activity but frequently correlates with modified expression of transcription factors and downstream pathway genes, indicating feedback regulation within the lignification network (Sattler et al., 2012).

### 3.2 Metabolic compensation and pathway plasticity

A distinguishing feature of *bmr* sorghum is the ability of the lignin biosynthesis network to partially offset enzymatic disturbances. In *bmr*6 (CAD-deficient) mutants, upstream aldehyde intermediates accumulate and integrate into the lignin polymer, leading to a structurally altered yet functionally adequate lignin matrix (Palmer et al., 2008). In *bmr*12 mutants, diminished COMT activity redirects monolignol flow towards guaiacyl-rich lignin while not entirely inhibiting polymer synthesis.

These compensatory strategies demonstrate metabolic adaptability in the phenylpropanoid system, where alternate enzymes, substrate promiscuity, and modified flux distributions sustain cell wall building. These reactions alleviate the severity of developmental anomalies and elucidate why partial lignin alteration can occur without detrimental effects on plant viability. The extent of compensation differs among *bmr* loci, resulting in locus-specific variations in agronomic performance and stress resilience.

### 3.3 Cell wall architecture and lignin-carbohydrate interactions

Lignin in grasses is intricately linked with hemicellulosic polysaccharides, especially arabinoxylans, resulting in complex lignin–carbohydrate complexes (LCCs) that significantly influence biomass recalcitrance. Ferulic acid residues esterified to arabinoxylans function as cross-linking agents, binding polysaccharide chains to lignin via oxidative coupling and strengthening the secondary cell wall matrix. Initial anatomical research suggested that enhanced digestibility in *bmr*12 sorghum is mostly linked to modified lignin distribution rather than significant disruption of cell wall structure (Akin et al., 1986).

Brown midrib (*bmr*) mutations alter both the chemical composition of lignin polymers and the distribution and quantity of lignin–carbohydrate cross-links. Decreased lignin content and modified monolignol composition in *bmr* sorghum are often associated with diminished ferulate-mediated cross-linking, leading to a more open cell wall structure and improved enzymatic accessibility. Microscopic and chemical investigations indicate that *bmr* stems possess thinner secondary walls, altered lignin deposition throughout wall layers, and enhanced wall porosity, all of which enhance digestibility and saccharification efficiency (Scully et al., 2016).

Notwithstanding the decrease in lignin concentration, the majority of *bmr* sorghum lines have functional vascular systems and sufficient mechanical support, underscoring the resilience of the SCW program. This resistance results from compensatory modifications in cellulose and hemicellulose deposition, along with qualitative alterations in lignin structure instead of complete lignin elimination. Excessive lignin reduction or significant process disruption can undermine wall integrity, resulting in phenotypes such as stem brittleness, increased lodging susceptibility, and reduced water transport.

The equilibrium between diminished recalcitrance and structural integrity is thus a pivotal factor in the application of *bmr* mutations. Among the identified loci, *bmr*12 and *bmr*19 typically achieve a beneficial equilibrium, but *bmr*2 and robust *bmr*6 alleles are more susceptible to adverse pleiotropic effects. These observations highlight the significance of optimizing lignin characteristics instead of striving for complete lignin suppression (Sattler et al., 2014; Saballos et al., 2009).

High-throughput transcriptome and metabolomic investigations have yielded comprehensive insights into the way *bmr* mutations alter cell wall production. The global expression analysis of *bmr* sorghum lines indicates a coordinated downregulation of lignin biosynthesis genes, along with modified expression of genes related to carbon metabolism, redox balance, and stress responses (Sattler et al., 2012). Metabolomic investigations indicate the buildup of phenylpropanoid intermediates and alterations in hydroxycinnamate pools, signifying modified route fluxes.

**Figure 2:**
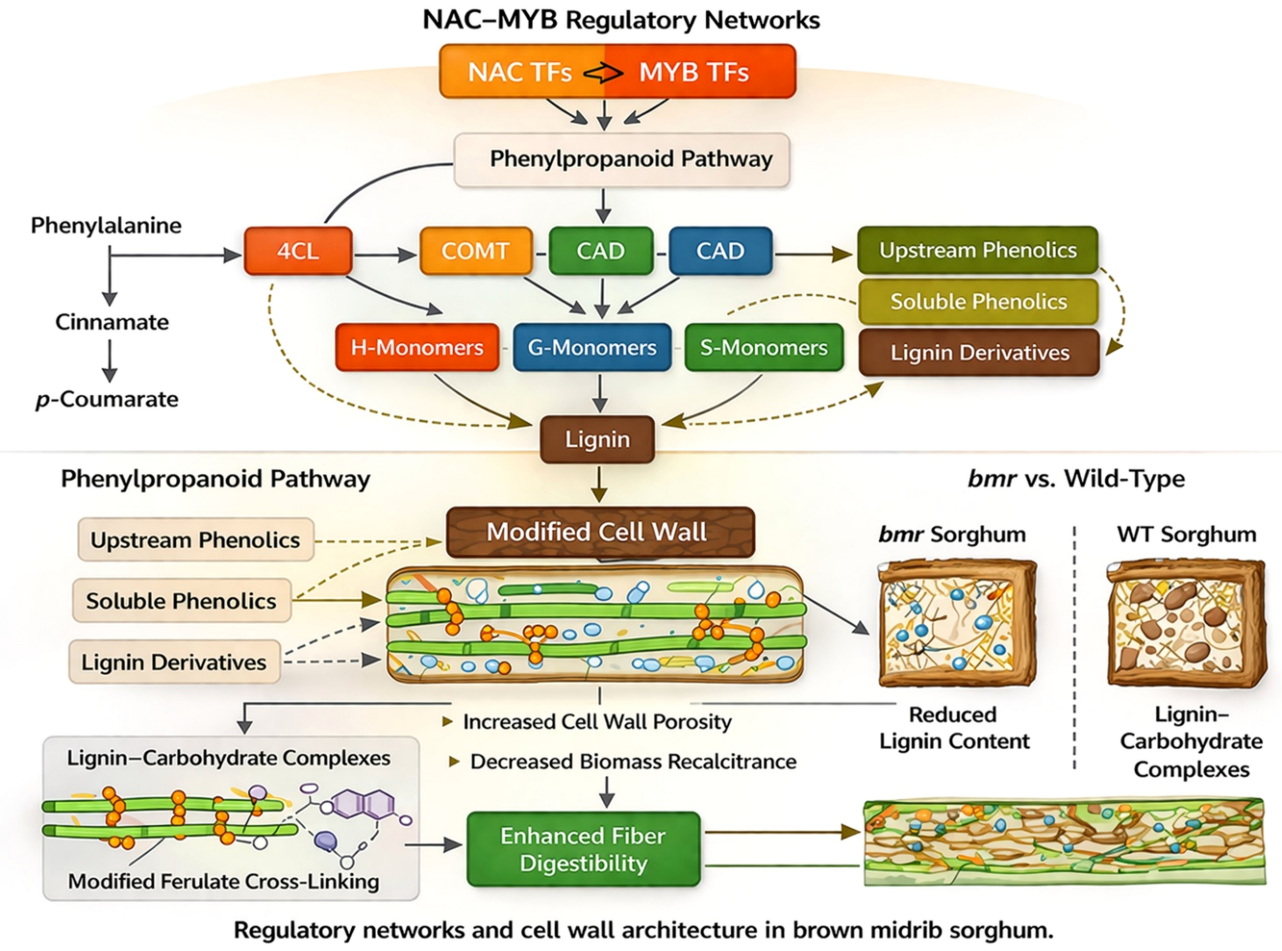
Regulatory framework linking phenylpropanoid metabolism to lignin deposition and cell wall properties in sorghum. This schematic illustrates how regulatory interactions control lignin biosynthesis in the phenylpropanoid pathway. It depicts enzymatic steps converting phenylalanine into lignin monomers, with key enzymes like 4-coumarate:CoA ligase (4CL), caffeic acid O-methyltransferase (COMT), and cinnamyl alcohol dehydrogenase (CAD). Upstream transcription factors from NAC and MYB families regulate these genes, affecting monolignol production, lignin polymerization, and lignin–carbohydrate complex formation in the secondary cell wall. Changes in lignin composition influence properties such as cell wall strength, digestibility, and biomass yield. The key enzymatic steps, initiated by phenylalanine, lead to the synthesis of H- and G-monolignols, which are then polymerized into lignin. This process is regulated by NAC–MYB networks. Variations in monolignol types and flow impact lignin amount, lignin–carbohydrate complexes, and ferulate cross-linking. These differences affect soluble phenolics, cell wall structure, and downstream traits like biomass yield, wall rigidity, and fiber digestibility. Dashed arrows indicate indirect or regulatory effects, while solid arrows show biochemical pathways.

These systemic responses underscore the interrelatedness of lignification with extensive metabolic networks and assert that lignin modification is more accurately perceived as a holistic system disruption rather than a singular pathway modification. Such discoveries are progressively shaping rational engineering strategies that focus on regulatory nodes rather than individual structural genes (**Figure 2**). The biochemical and molecular insights derived from *bmr* mutants ultimately have practical implications for crop performance. Alterations in lignin content and composition influence not only biomass processability but also plant growth, structural stability, and interactions with environmental stresses.

### 3.4 Agronomic performance and trade-offs of *bmr* sorghum

Brown midrib (*bmr*) mutations are acknowledged for enhancing cell wall digestibility and diminishing biomass recalcitrance; nevertheless, their impact on agronomic performance is intricate and locus-specific. Lignin is essential for mechanical strength and vascular integrity; hence, its alteration frequently leads to diverse impacts on plant growth, biomass accumulation, and stability. Field tests of *bmr* sorghum repeatedly indicate that decreased lignin concentration may correlate with yield decreases, especially in high biomass production scenarios (Casler and Vogel, 1999; Dien et al., 2009).

The *bmr*6 and *bmr*2 mutants, among characterized loci, often display diminished plant height, slender stems, and decreased total dry matter yield relative to wild-type variants. These effects result from weakened secondary cell wall integrity and modified carbon allocation, which may restrict vascular efficiency and structural support. Conversely, *bmr*12 and *bmr*19 mutants frequently exhibit nearly normal growth and biomass accumulation, underscoring the significance of route location and metabolic integration in influencing agronomic results (Scully et al., 2016; Adeyanju et al., 2021).

### 3.5 Lodging disease resistance, and environmental interactions

Lodging constitutes a major agronomic challenge linked to *bmr* sorghum. Decreased lignin content compromises sclerenchyma tissues and diminishes stem bending strength, heightening susceptibility to lodging caused by wind and rain. Empirical studies indicate that *bmr*6 sorghum lines are especially susceptible to lodging, particularly under conditions of high nitrogen fertilizer or dense planting that encourage rapid vegetative growth (Saballos et al., 2008; Sattler et al., 2010a).

Nonetheless, lodging susceptibility is not an unavoidable result of lignin alteration. The genetic background, stem diameter, internode length, and overall plant architecture significantly influence the risk of lodging. Breeding strategies that integrate *bmr* alleles into resilient, thick-stemmed genotypes have effectively alleviated lodging penalties, illustrating that structural compensation can partially counterbalance diminished lignification. These findings underscore the necessity of assessing *bmr* lines under authentic agronomic circumstances instead of depending exclusively on controlled-environment evaluations.

Lignin serves as an essential physical and chemical barrier against pathogen invasion, and its alteration can affect disease susceptibility (Miedes et al., 2014; Bonawitz and Chapple, 2013). Numerous studies indicate heightened susceptibility of *bmr* sorghum to stalk rots and foliar diseases, such as charcoal rot (*Macrophomina phaseolina)* and *Fusarium*-related infections (Khasin et al., 2021: Bandara et al., 2018). Decreased lignin accumulation may enable pathogen entry and undermine structural barriers in vascular tissues.

Nonetheless, disease responses in *bmr* genotypes exhibit considerable variability, contingent upon the precise mutation and the pathogen implicated. Certain *bmr*12 lines demonstrate disease resistance akin to non-*bmr* controls, indicating that qualitative alterations in lignin composition may maintain defensive capabilities despite a reduction in total lignin content (Sattler and Funnell-Harris, 2013). These data highlight the necessity of incorporating disease resistance features in *bmr* breeding programs to prevent inadvertent susceptibility. Decreased lignification may affect disease susceptibility by compromising structural and chemical defenses (**Table 3**).

**Table 3:** Disease responses and pathogen susceptibility associated with brown midrib (*bmr*) backgrounds in cereals.

Pathogen	Disease	Evidence in *bmr* backgrounds
*Macrophomina phaseolina*	Charcoal rot	Reduced lignin + drought synergistically increases susceptibility: *bmr*6 and *bmr*12 sorghum exhibit longer lesions under severe stress.
*Fusarium* spp.	Fusarium stalk rot	*bmr* maize lines generally show increased susceptibility due to weaker rind strength.
*Claviceps, Colletotrichum*	Occasionally affected	Lignin mutants sometimes show higher susceptibility but results vary with genotype and environment.

Environmental factors significantly affect the manifestation of *bmr* phenotypes. Water availability, temperature, soil fertility, and planting density can influence lignin deposition and cell wall composition, consequently impacting the extent of *bmr*-related characteristics (Van Acker et al., 2013; Christensen and Rasmussen, 2019). During drought stress, sorghum often enhances lignin deposition to preserve vascular function, potentially offsetting the lignin reductions caused by *bmr* (Khasin et al., 2021).

Field trials in various habitats indicate that yield penalties linked to *bmr* mutations are frequently less significant under stress situations, where less recalcitrance may provide benefits by enhancing carbon use efficiency and resource distribution (Christensen and Rasmussen, 2019). These genotype-by-environment interactions underscore the adaptive plasticity of lignification and underscore the necessity for multi-location trials to precisely evaluate *bmr* function.

The effective implementation of *bmr* sorghum depends on reconciling enhanced biomass usage with satisfactory agronomic performance. Breeding strategies designed to alleviate trade-offs encompass the utilization of partial-loss-of-function alleles, the pyramiding of *bmr* loci with synergistic effects, and the introgression into superior genetic backgrounds characterized by robust stem architecture. Reduced lignin in *bmr* mutants improves cell wall digestibility and biomass conversion efficiency, making them valuable targets for breeding programs (Sattler et al., 2014; Li et al., 2015). Marker-assisted selection and genomic selection have expedited the identification of *bmr* lines exhibiting advantageous trait combinations (Baloch et al., 2023).

Recent studies indicate that integrating *bmr* mutations with characteristics like diminished plant height, increased stem thickness, or optimized root architecture can significantly mitigate lodging risk while maintaining improvements in digestibility (Umakanth et al., 2014). These integrated breeding strategies illustrate that lignin alteration can be achieved without compromising field performance when informed by a comprehensive understanding of plant structure and physiology.

From a practical standpoint, the agronomic efficacy of brown midrib (*bmr*) sorghum should be assessed about its intended application. Decreases in lignin linked to *bmr* mutations frequently improve cell wall digestibility in cereal grasses (Sattler et al., 2010b). In forage systems, little reductions in biomass yield are frequently counterbalanced by significant improvements in digestibility, voluntary consumption, and the production of milk or meat.

In bioenergy systems, enhanced saccharification efficiency and diminished pretreatment severity can offset reduced biomass yields, resulting in advantageous overall energy balances and better economic viability (Dien et al., 2009; Scully et al., 2016). Thus, the agronomic trade-offs linked to *bmr* mutations should be regarded not as intrinsic limitations, but as manageable constraints that can be alleviated by focused breeding, suitable genetic backgrounds, and enhanced crop management approaches. In several studies and grass species, *bmr* mutations reliably decrease lignin concentration, while demonstrating agronomic impacts that are depending on the locus and genetic background (**Table 4**).

**Table 4:** Effects of brown midrib (*bmr*) mutations on lignin composition and agronomic performance in sorghum and grasses.

Study (Author, Year)	Crop/allele	Lignin change (reported)	Primary compositional change	Agronomic/other notes
Saballos et al., 2009	Sorghum *bmr*2, *bmr*6, *bmr*12	Lignin ↓ (~10–35%)	Altered CAD/COMT-related S: G and cross-linking	Improved digestibility; background-dependent yield effects.
Emendack et al., 2022	*bmr*12 mutants	reduced lignin	—	New germplasm for testing.
van der Weijde et al., 2013	Review (maize & grasses)	*bmr* reduces lignin	Composition shifts noted	Discussion of conversion benefits vs. agronomic trade-offs.

## 4 Applications of *bmr* sorghum in forage and bioenergy systems

### 4.1 Forage quality and digestibility improvements

Forage digestibility is a crucial factor influencing ruminant production and feed efficiency, with lignin being acknowledged as the most restrictive element of plant cell walls in this regard. In contrast to cellulose and hemicellulose, lignin is fundamentally indigestible by rumen microbes and creates a physical barrier that limits microbial access to fermentable polysaccharides. In sorghum, elevated lignin levels and significant lignin–carbohydrate cross-linking result in reduced neutral detergent fiber digestibility (NDFD) compared to other forage crops, hence limiting its nutritional value despite substantial biomass yield.

Brown midrib (*bmr*) mutations directly mitigate this constraint by decreasing lignin content and modifying lignin composition, leading to secondary cell walls that are more susceptible to microbial breakdown. Initial ultrastructural and digestibility investigations revealed that the *bmr*12 mutation significantly improves tissue degradability compared to standard sorghum varieties. Akin et al. (1986) demonstrated using microscopic and *in vitro* digestion methods that diminished lignification in *bmr*12 plants enhanced microbial accessibility to secondary cell walls, especially in extensively lignified vascular and sclerenchyma tissues. Significantly, enhanced digestibility correlated with modified lignin deposition and wall organization rather than substantial anatomical anomalies, offering preliminary evidence that specific alterations in lignin structure and distribution might markedly enhance fodder utilization.

A recent quantitative synthesis of forage research has yielded substantial, population-level evidence for the nutritional benefits of *bmr* sorghum. Using a meta-analysis approach, Widodo et al. (2025) demonstrated that brown midrib sorghum consistently exhibits lower lignin concentration and higher fiber digestibility than conventional sorghum across diverse environments and management conditions. Notable enhancements in NDFD and crude protein utilization demonstrated that the forage quality advantages of *bmr* sorghum are consistent rather than unique to individual studies. These data collectively demonstrate that *bmr* sorghum is an excellent method for enhancing feed quality and underscore its significance as a dual-purpose crop for livestock production and bioenergy systems. These findings illustrate that the practical value of *bmr* sorghum in forage systems arises from improvements in feed efficiency and livestock productivity, even when moderate reductions in biomass yield occur.

Comprehensive feeding trials and *in vitro* digestion assays consistently indicate that *bmr* sorghum possesses markedly superior fiber digestibility compared to non-*bmr* varieties. Decreases in acid detergent lignin (ADL) and alterations in lignin structure result in enhancements in *in vitro* dry matter digestibility (IVDMD) and neutral detergent fiber digestibility (NDFD), typically ranging from 5 to 15 percentage points, contingent upon the specific *bmr* locus and genetic background (Sattler et al., 2010b). Among the identified loci, *bmr*12 and *bmr*6 exhibit notably enhanced digestibility. Lines with reduced syringyl lignin production demonstrate elevated rates of fiber breakdown due to diminished polymer condensation. *Bmr*6 mutants, distinguished by aldehyde-rich lignin, exhibit heightened vulnerability to microbial invasion, however structural deficiencies may restrict their agricultural application. These disparities highlight the significance of lignin composition, rather than only lignin quantity, in assessing fodder quality.

Integrating brown midrib (*bmr*) alleles into forage sorghum breeding has been extensively studied to improve feed quality for ruminants. Among the known loci, *bmr*6 and *bmr*12 are most commonly used because they strongly reduce lignin levels and improve fiber digestibility. Field tests show that sorghum lines with these alleles consistently achieve better neutral detergent fiber digestibility (NDFD) and *in vitro* dry matter digestibility (IVDMD) than traditional sorghum varieties (Soufizadeh et al., 2018; Holman et al., 2018; Adeyanju et al., 2021). These improvements result in higher voluntary intake and enhanced productivity in feeding trials with dairy cattle and beef steers. Multiple studies indicate that *bmr* sorghum silage can partly replace maize silage in dairy diets without reducing milk yield or feed efficiency. Nonetheless, the performance of *bmr*-containing sorghum can vary with genetic background, environmental factors, and the specific mutation. Breeding efforts, therefore, focus on combining *bmr* alleles with elite germplasm that has strong stem structure and lodging resistance to maintain field robustness while enhancing forage quality.

### 4.2 Livestock performance and silage characteristics

**Figure 3:**
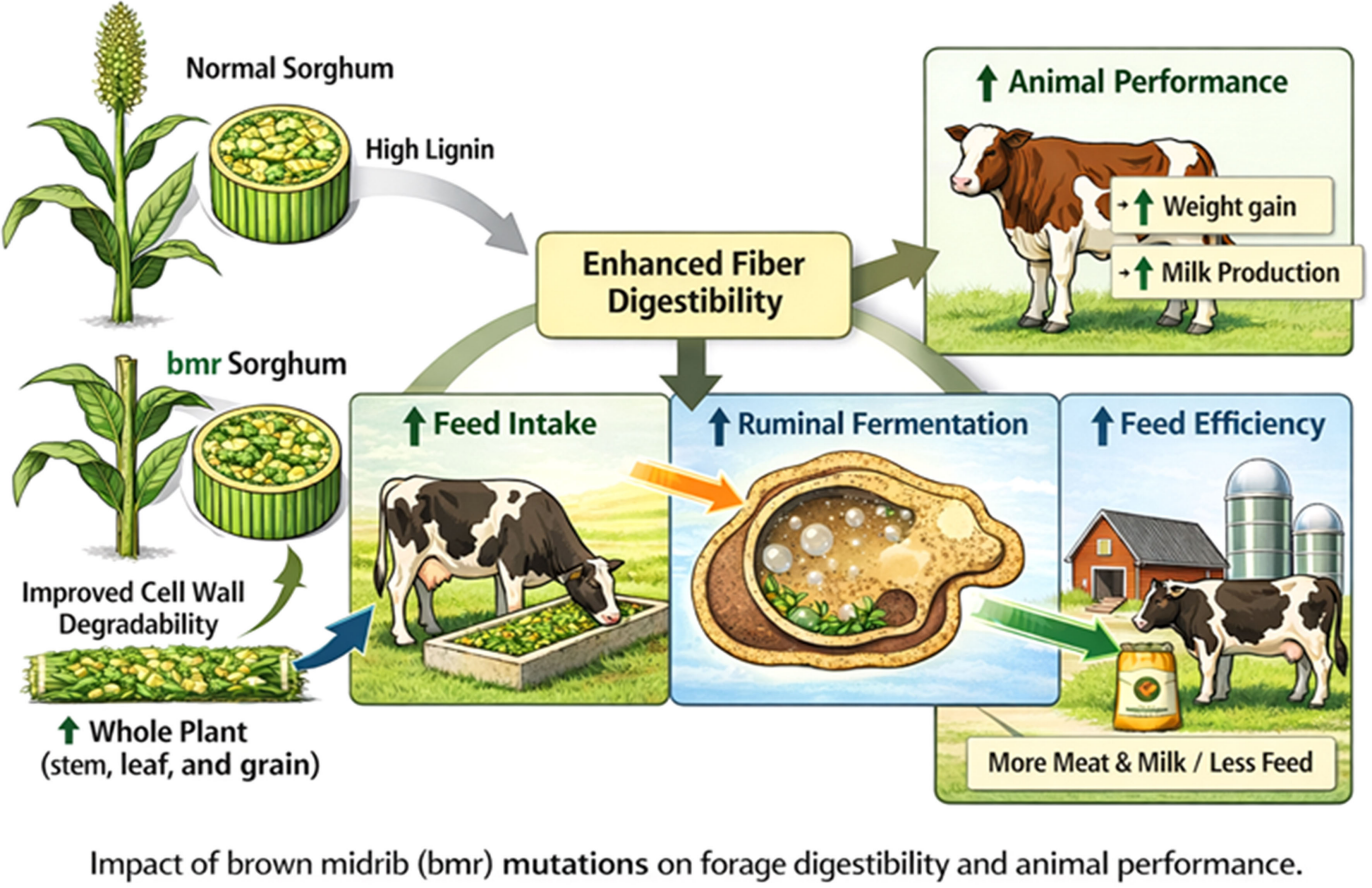
Relationship between lignin modification in brown midrib sorghum and improved forage utilization in ruminant livestock.*bmr* The diagram illustrates how reducing lignin content and changing its composition in *bmr* sorghum improves cell wall breakdown and microbial access in the rumen. These adjustments enhance fermentation, increase nutrient availability, and stimulate voluntary feed intake. Such improvements, proven through forage sorghum feeding studies, lead to higher livestock productivity, including increased milk yield and weight gain. This framework is based on research findings from forage sorghum and livestock trials, rather than just a conceptual model. Additionally, **Figure 3** depicts how lowering lignin in sorghum improves fiber digestibility, ruminal fermentation, and nutrient availability, which in turn boosts feed efficiency, reduces methane emissions, and enhances performance traits like body weight gain and milk production. Overall, it emphasizes the pathway from modified sorghum biomass to increased livestock productivity and sustainability.

Enhanced fiber digestibility in *bmr* sorghum directly affects voluntary feed intake and rumen fermentation dynamics. The reduced lignin content of *bmr* forages improves cell-wall degradability and increases ruminal digestion of neutral detergent fiber (NDF), which enhances nutrient availability for ruminants (Ferreira et al., 2018). Improved cell wall degradability accelerates passage velocity and diminishes rumen fill limitations, resulting in elevated dry matter intake (DMI) by ruminants. Feeding trials with dairy cattle and beef steers consistently indicate elevated dry matter intake when *bmr* sorghum silage or hay is included in the diet, compared with standard sorghum forages (Oliver et al., 2004). The relationship between lignin modification in brown midrib sorghum and improved ruminant feed utilization has been demonstrated in forage feeding studies where both normal and *bmr* sorghum are typically harvested and fed as whole-plant forage containing stems and leaves. This relationship is summarized in **Figure 3**.

An elevated intake and optimized rumen fermentation result in superior animal performance indicators, such as augmented milk production, enhanced feed conversion efficiency, and increased weight gain. *bmr* sorghum silage can partially replace maize silage in dairy systems without diminishing milk output, providing a drought-resistant option in water-scarce areas (Ferreira et al., 2018). The performance advantages underscore the capacity of *bmr* sorghum to enhance animal productivity while ensuring sustainability. Comparative feeding trials indicate that *bmr* sorghum silage nearly matches maize silage in terms of digestibility and animal performance (**Table 5**).

**Table 5:** Effects of brown midrib (*bmr*) sorghum silage on forage quality, resource use, intake, and milk production compared with conventional sorghum and maize silage.

Trait/Parameter	Normal sorghum silage	*bmr* sorghum silage	Maize silage	Key interpretation
Lignin content	Moderate–high	Reduced (*bmr*-specific)	Low	*bmr* mutation compensates for sorghum’s inherently higher lignin
NDF digestibility (%)	45–50	55–65	60–65	*bmr* sorghum approaches maize-level fiber digestibility
Dry matter intake (DMI)	Baseline	+1.0–2.0 kg day^−1^	High	Improved digestibility drives higher voluntary intake
Milk yield (dairy cows)	Baseline	+1.0–2.5 kg day^−1^	High	Milk yield response often comparable to maize silage
Milk fat %	Normal	Stable or slightly increased	Stable	No negative effect of *bmr* forage on milk quality
Feed efficiency	Baseline	Improved	High	Higher milk output per unit DMI
Small ruminant response	Moderate	+10–20% milk yield (goats); ↑ growth (sheep)	Limited data	*bmr* sorghum improves intake and digestibility in goats and sheep
Water requirement	Lower than maize	Low	High	*bmr* sorghum advantageous in water-limited systems
Heat tolerance	High	High	Low–moderate	*bmr* sorghum better suited to tropical and semi-arid regions
Overall suitability	Moderate	High (dual-purpose forage)	High (resource-intensive)	*bmr* sorghum offers maize-like performance with lower resource demand

*bmr* mutations affect the kinetics and quality of silage fermentation, in addition to digestibility. Decreased lignin content accelerates fermentation by lactic acid bacteria, resulting in a more rapid pH decrease and enhanced silage preservation. Several studies report lower neutral detergent fiber concentrations and improved fermentation profiles in *bmr* sorghum silage, characterized by higher lactic acid content and reduced proteolysis (Oliver et al., 2004; Kung et al., 2018).

Nonetheless, enhanced digestibility may potentially heighten the risk of silage effluent losses and aerobic instability if inadequately controlled (Kung et al., 2018). To effectively capitalize on the advantages of *bmr* sorghum in forage systems, it is imperative to optimize harvesting time, moisture content, and ensiling procedures.

Improved forage quality in brown midrib (*bmr*) sorghum often correlates with slight decreases in biomass output, especially in genotypes exhibiting significant lignin reduction. This trade-off prompts significant inquiries concerning net feed value per unit of land area. Numerous studies demonstrate that improvements in fiber digestibility and voluntary intake can compensate for reduced biomass output, leading to comparable or enhanced animal productivity per hectare relative to non-*bmr* sorghum (Casler and Vogel, 1999).

Alterations in lignin production may affect plant defense mechanisms. Sattler and Funnell-Harris (2013) conducted a thorough evaluation of the implications of lignin reduction or compositional modification on plant–pathogen interactions, highlighting that lignin serves both as a structural element and as a barrier to biotic stress. Their investigation revealed that whereas *bmr* mutations often enhance biomass digestibility and conversion efficiency, disease responses are significantly context-dependent and differ according to the individual lignin biosynthetic step impacted. These findings highlight the necessity of balancing diminished cell wall recalcitrance with the preservation of biotic stress resistance when utilizing *bmr* sorghum, especially in bioenergy production systems.

From a systems perspective, the agronomic value of *bmr* sorghum is most accurately evaluated based on animal production rather than solely on biomass yield. Within this comprehensive framework, *bmr* sorghum often surpasses traditional varieties in forage-based livestock systems, particularly in stress-prone settings where sorghum’s natural resilience offers a competitive edge.

The implementation of *bmr* sorghum is consistent with the objectives of sustainable livestock production. Enhanced digestibility increases feed efficiency and diminishes methane emissions per unit of animal product, hence reducing environmental footprints. Moreover, the drought and high-temperature endurance of *bmr* sorghum variants renders them especially advantageous in light of climate change and escalating resource limitations (Widodo et al., 2025; Blaxter and Clapperton, 1965). Integrating genetic alteration of lignin with suitable agronomic management and ration formulation, *bmr* sorghum presents a feasible approach to enhancing robust and efficient forage systems that fulfill productivity and sustainability objectives (**Figure 3**). Feeding trials conducted in various agro-climatic regions further validate the consistent advantages of *bmr* sorghum feed (**Table 6**).

**Table 6:** Effect of *bmr* sorghum forage on milk production and performance of ruminant livestock: evidence from Indian and global feeding trials.

Region	Animal	Forage type	Key observations	Milk yield/performance effect	Reference
USA/India	Lactating cows	*bmr* sorghum silage (*bmr*6, *bmr*12)	Higher NDF digestibility and intake	+1.5–2.5 kg milk day^−1^	Oliver et al., 2004; Widodo et al., 2025; Rao et al., 2012
USA	Lactating cows	*bmr* sorghum silage	Increased DMI and feed efficiency	Milk yields comparable to maize silage	Aydin et al., 1999
USA/Italy/Brazil	Lactating cows	*bmr* sorghum vs maize silage	Improved fiber digestibility	No negative effect on milk fat	Grant and Ferraretto, 2018; Tudisco et al., 2021

Across different agro-climatic regions, feeding trials consistently show that *bmr* sorghum forage enhances fiber digestibility, voluntary intake, and milk production in ruminants, with performance often like that of maize silage, especially under heat and water-stress conditions.

### 4.3 Bioethanol production and sacharification efficiency

Lignocellulosic biomass serves as a renewable and plentiful feedstock for sustainable biofuel production; nevertheless, its conversion efficiency is limited by intrinsic biomass recalcitrance. Lignin is the primary factor contributing to this limitation because of its aromatic complexity, hydrophobic nature, and significant cross-linking with polysaccharides, which collectively hinder the enzymatic hydrolysis of cellulose and hemicellulose (Himmel et al., 2007). In grasses like sorghum, recalcitrance is further enhanced by ferulate-mediated lignin–carbohydrate complexes that fortify the secondary cell wall matrix. The structural variability of native lignin limits biological upgrading, emphasizing the necessity for feedstocks with altered lignin chemistry to enhance processing efficiency (Beckham et al., 2016).

Brown midrib (*bmr*) sorghum offers a genetically manipulable framework for implementing these alterations. Comparative analyses indicate that *bmr* lignocellulosic tissues demonstrate less recalcitrance, hence improving fodder digestibility and bioenergy conversion efficiency (Sattler et al., 2010a). Strategies that diminish lignin content or adjust lignin composition—such as designing one-carbon metabolism to limit S-adenosylmethionine (SAM)-dependent methylation—can further alter lignin structure and enhance saccharification efficiency in sorghum (Tian et al., 2024).

The amalgamation of *bmr* genetics with chemical and biological funneling methodologies illustrates the significant impact of lignin structure on subsequent biomass valorization (Linger et al., 2014). These characteristics render *bmr* sorghum a compelling feedstock for bioethanol production and advanced biofuel systems, optimizing processability alongside elevated biomass productivity.

Pre-treatment is an essential phase in lignocellulosic biofuel production, aimed at altering cell wall architecture and improving enzyme accessibility. Nonetheless, pre-treatment techniques frequently entail significant energy consumption, harsh chemical conditions, and substantial costs. Multiple studies indicate that *bmr* sorghum necessitates less rigorous pretreatment conditions than non-bmr biomass to attain similar or superior sugar release (Dien et al., 2009; Scully et al., 2016) (**Table 7**). The *bmr*6 and *bmr*12 lines demonstrate decreased lignin content and modified monomer composition, which impair lignin–carbohydrate interactions and enhance delignification after pretreatment. Consequently, diminished temperatures, decreased acid or alkali contents, and abbreviated residence periods are adequate for successful biomass decomposition. These enhancements result in decreased processing expenses and diminished environmental effect, hence bolstering the economic viability of sorghum-based biorefineries.

**Table 7:** Effects of brown midrib (*bmr*)–associated lignin modification on biomass pretreatment and biofuel conversion efficiency.

Study (Author, Year)	Pretreatment	Enzyme load	Saccharification outcome (vs. WT)	Ethanol/fermentation outcome	Notes/caveats
Dien et al., 2009	Mild alkaline (NaOH)	Standard cellulase cocktail	Sugar yields ↑ (often 15–40%)	Ethanol yields ↑ (~10–25%)	Varies by *bmr* allele and background.
Chen and Dixon, 2007	Acid/enzymatic comparisons	Varied	Improved fermentable sugar yields for low-lignin feedstocks	Improved conversion efficiency reported	Focused on mechanistic links.
Kim et al., 2012	Fast pyrolysis	n/a	Differential pyrolysis product distribution	Impacts on bio-oil composition	Lower lignin can change energy density.

Post-treatment, the efficiency of enzymatic hydrolysis is a critical factor influencing the total biofuel production. *bmr* sorghum regularly exhibits superior enzymatic saccharification efficiency compared to conventional sorghum, as evidenced by enhanced glucose and xylose release from both stem and leaf tissues. Improvements in sugar yields have been reported to vary from 20% to over 40%, contingent upon the specific *bmr* allele, pre-treatment method, and enzyme cocktail employed (Dien et al., 2009). Significantly, enhancements in saccharification efficiency cannot be exclusively ascribed to diminished lignin content. Structural changes to lignin, such as reduced polymer condensation and modified interunit links, improve enzyme accessibility. These findings underscore the importance of focusing on lignin quality instead of engaging in indiscriminate lignin reduction, which may jeopardize plant fitness.

The increased sugar release from *bmr* sorghum biomass immediately results in elevated bioethanol yields during fermentation. Comparative analyses utilizing yeast and bacterial fermentation systems consistently demonstrate enhanced ethanol production per unit biomass for *bmr* lines compared to wild-type controls (Dien et al., 2009). In many instances, ethanol yields from *bmr*12 sorghum are comparable to or surpass those derived from maize stover under analogous processing settings. In addition to yield enhancements, *bmr* sorghum provides further benefits for bioethanol production, such as decreased inhibitor development during pre-treatment and enhanced fermentation kinetics. Reduced amounts of phenolic inhibitors emanating from changed lignin structures can augment microbial function, hence diminishing the necessity for detoxification procedures and enhancing overall process efficiency. **Table 8** elucidates field data for enhanced saccharification and ethanol production in brown midrib (*bmr*) sorghum.

**Table 8:** Experimental and field evidence for improved saccharification and ethanol production in brown midrib (*bmr*) sorghum.

Paper (year)/species	*bmr* allele(s)	Lignin change (reported)	Saccharification/glucose ↑	Ethanol yield/fermentation result	Notes/caveats
Dien et al. (2009). *forage sorghum*	*bmr*-6, *bmr*-12, double	reduced lignin (allele dependent)	Glucose yields ↑: *bmr*-6 = 27%, *bmr*-12 = 23%, double = 34% vs WT (after pretreatment + enzymatic hydrolysis).	Ethanol yields increased in line with released sugars (improved ethanol yield per unit biomass).	Classic, highly cited experimental demonstration for sorghum *BMR* → better sugar release/ethanol in lab assays.
Guragain et al. (2014), *low-lignin mutants*	various low-lignin mutants	lower total lignin, but composition matters	saccharification improved in many cases (varies by genotype)	Ethanol yield from released glucose ≈ >95% theoretical (i.e., fermentation not limiting).	Shows that once sugars are released, fermentation is efficient; the main issue is releasing those sugars.
Sattler et al. (2010b) — *maize, sorghum, pearl millet*	review of *bmr* alleles	summarizes consistent lignin reductions across *BMR* types	reports increased conversion rates and improved saccharification in many studies	notes benefits for bioenergy but highlights genotype × environment and yield tradeoffs.	Important review for mechanistic background and cautionary points.
Rivera-Burgos et al. (2019). *brown-midrib sweet sorghum*	*bmr* in sweet sorghum	confirms reduced lignin in *bmr* sweet sorghum	Theoretical ethanol production increased because of higher stover sugar potential and lower lignin	recommends selecting high-yielding *bmr* backgrounds to avoid biomass penalties.	Good recent dataset across 236 RILs; addresses breeding potential for dual-purpose lines.

### 4.4 Advanced biofuels and biorefinery applications

Besides traditional bioethanol, brown midrib (*bmr*) sorghum is ideally suited for advanced biofuel processes, encompassing the production of butanol, renewable hydrocarbons, and bio-based compounds. Improved saccharification efficiency enhances the accessibility of fermentable sugars for various microbial platforms, facilitating adaptable biorefinery setups. Sweet sorghum provides many bioenergy pathways and exhibits great resource-use efficiency, establishing it as a robust foundation for lignin-optimized bioenergy feedstocks (Regassa and Wortmann, 2014). Furthermore, modified lignin from *bmr* sorghum may exhibit enhanced susceptibility to depolymerization and valorization, hence facilitating the production of lignin-derived coproducts such as aromatics, resins, and carbon fibers (Ragauskas et al., 2014).

Biological hydrogen production from lignocellulosic biomass is significantly hindered by cell wall recalcitrance, as lignin content and composition restrict both sugar liberation and microbial accessibility. Bhatia et al. (2022) underscored that efficient pre-treatment methods, along with feedstocks that have inherently diminished or altered lignin, are essential for improving biohydrogen production and overall process efficacy. Their bio-economic research emphasized that the incorporation of low-recalcitrance biomass and the valorization of residual lignin streams are crucial for attaining techno-economic viability in future biohydrogen biorefineries.

The integration of *bmr* sorghum into biorefineries demonstrates comprehensive biomass use, where both carbohydrate and lignin components are effectively transformed into value-added products. Comprehensive strategies are essential for enhancing the economic feasibility and sustainability of lignocellulosic biofuel systems.

### 4.5 Sustainability and life-cycle implications

*bmr* sorghum presents numerous benefits compared to conventional bioenergy feedstocks from a sustainability standpoint. Its elevated water-use efficiency, drought resilience, and adaptability to suboptimal lands diminish competition with food crops and mitigate environmental repercussions. Life-cycle studies demonstrate that enhanced conversion efficiency in *bmr* sorghum can diminish greenhouse gas emissions per biofuel unit produced by decreasing energy inputs and chemical utilization during processing (Scully et al., 2016). *bmr* sorghum, when integrated with sustainable agronomic practices and biorefinery designs, can significantly enhance renewable energy portfolios and bolster climate-resilient agricultural systems.

## 5 Systems biology and genome editing for lignin engineering

### 5.1 Multi-omics and regulatory network reconstruction

Initial investigations on brown midrib (*bmr*) sorghum predominantly utilized phenotypic characterization and specific biochemical experiments, concentrating on individual enzymes in the lignin synthesis pathway. Although these methods produced essential insights into monolignol production, they provided insufficient understanding of how lignin alteration influences broader metabolic and regulatory networks. Systems biology approaches have transformed lignin research by enabling integrative analysis of transcriptomic, proteomic, metabolomic, and phenomic datasets. Rather than simply cataloguing pathway components, these approaches allow identification of regulatory hubs controlling lignification. For example, transcriptomic analyses of sorghum *bmr* mutants have revealed coordinated regulation of NAC–MYB transcription factor networks together with genes involved in carbon allocation, redox homeostasis, and phenylpropanoid metabolism. Such findings demonstrate that lignin modification triggers broader metabolic reprogramming rather than isolated pathway perturbations (Sattler et al., 2012; Vanholme et al., 2019).

In *bmr* sorghum, systems-level investigations indicate that lignin alteration initiates extensive transcriptional reprogramming outside the phenylpropanoid pathway, influencing genes related to carbon allocation, redox balance, hormone signaling, and stress responses. These findings highlight the interrelatedness of lignification with total plant metabolism and development. These findings highlight that lignin engineering should be approached as a systems-level challenge involving coordinated regulation of metabolic flux, transcriptional networks, and cell wall assembly rather than simple manipulation of individual structural genes.

### 5.2 Transcriptomics, metabolomics, and proteomics insights

RNA sequencing-based transcriptomic profiling has proven crucial in clarifying the regulatory effects of *bmr* mutations. Comparative analyses of *bmr* and wild-type sorghum lines consistently demonstrate coordinated downregulation of lignin biosynthetic genes, alongside modified expression of transcription factors implicated in secondary cell wall formation, particularly members of the NAC and MYB families (Sattler et al., 2012).

The intricacy of lignin transcriptional networks suggests that successful lignin engineering must consider regulatory hierarchy and compensating mechanisms (Zhao and Dixon, 2011). Network reconstruction methodologies have pinpointed essential regulatory hubs that amalgamate developmental signals with lignin production. These hubs are appealing targets for the indirect modification of lignin characteristics, as slight alterations in regulatory genes can precisely adjust lignin deposition without inducing significant developmental anomalies. Moreover, transcriptome analyses underscore genotype-specific responses, underscoring the significance of genetic background in interpreting *bmr*-related regulatory alterations.

Metabolomic investigations provide mechanistic insights into how *bmr* mutations redirect metabolic flux through the phenylpropanoid pathway. Profiling studies in *bmr*6 and *bmr*12 sorghum lines have shown accumulation of upstream intermediates such as cinnamaldehydes and hydroxycinnamic acids, together with altered pools of soluble phenolics. These metabolic signatures indicate enzymatic bottlenecks and compensatory redirection of carbon into flavonoid and hydroxycinnamate pathways. Such metabolic plasticity highlights the capacity of sorghum to maintain cell wall integrity despite partial disruption of monolignol biosynthesis.*bmr* The profiling of phenylpropanoid intermediates in *bmr* sorghum has demonstrated the accumulation of upstream substrates, depletion of downstream monolignols, and alterations in hydroxycinnamate conjugates, indicating altered enzymatic bottlenecks (Palmer et al., 2008; Adeyanju et al., 2021).

Stable isotope labeling and flow analysis techniques further illustrate that lignin modification influences carbon distribution between structural and soluble phenolics. These alterations can affect stress responses and the synthesis of defense-related metabolites, offering mechanistic insights into the reported trade-offs in disease resistance and environmental resilience. Metabolomics and flux analysis enhance understanding of the biochemical ramifications of lignin engineering and guide approaches to mitigate adverse effects.

Although transcriptomic and metabolomic analyses have significantly advanced our understanding of lignin biosynthesis, proteomic studies are beginning to reveal additional layers of regulation at the post-translational level. Protein abundance, enzyme localization, and post-translational modifications such as phosphorylation can influence metabolic pathway activity independently of transcript abundance. Proteomic investigations in plants have demonstrated that differential accumulation of enzymes involved in lignin biosynthesis and related pathways can regulate lignin deposition and plant defense responses (Wang et al., 2020).

In sorghum, mutations affecting lignin biosynthesis genes such as cinnamyl alcohol dehydrogenase (CAD) and caffeic acid O-methyltransferase (COMT)—associated with the *bmr*6 and *bmr*12 phenotypes—alter enzyme expression patterns and cell-wall phenolic composition, indicating that changes at the protein level can significantly influence lignification processes (Li et al., 2015; Khasin et al., 2021).

In addition, oxidative enzymes such as laccases, which catalyze the polymerization of monolignols during lignin formation, show variable expression and potential post-translational regulatory sites in sorghum, suggesting complex regulation of lignin polymerization beyond gene transcription alone (Wang et al., 2017).*bmr*Integrating proteome data with transcriptome and metabolomic profiles is crucial for developing predictive models of lignification and for identifying regulatory leverage points that can be targeted through breeding or biotechnology.

### 5.3 Genome editing as a next-generation bmr strategies

Genome editing technologies, particularly CRISPR/Cas systems, provide powerful tools for precise manipulation of lignin biosynthesis genes in sorghum. Unlike classical *bmr* mutants, which often involve complete loss-of-function mutations, genome editing enables targeted modification of specific catalytic domains, promoter elements, or regulatory motifs to fine-tune enzyme activity. For instance, editing of COMT, CAD, and CCR genes can generate partial-loss-of-function alleles that reduce lignin recalcitrance while preserving sufficient lignification for structural integrity. Emerging strategies also target transcriptional regulators, such as NAC and MYB transcription factors, to modulate lignin deposition at the transcriptional level. In contrast to standard *bmr* mutants, which typically result in a total or near-total loss of gene function, genome editing permits the precise alteration of specific domains, regulatory regions, or alleles to achieve partial decreases in enzyme activity (Huang et al., 2026; Liu et al., 2019).

In sorghum, CRISPR/Cas-mediated editing of lignin biosynthesis genes, including COMT (caffeic acid O-methyltransferase), CAD (cinnamyl alcohol dehydrogenase), and CCR (cinnamoyl-CoA reductase), has been explored to generate targeted *bmr*-like phenotypes. These modifications typically aim to reduce lignin content or alter lignin composition, thereby improving cell wall digestibility and biomass saccharification efficiency. In contrast to classical *bmr* mutants, genome editing enables the generation of precisely tuned alleles, including partial-loss-of-function variants that minimize negative effects on plant growth or structural integrity. Reported phenotypes include altered lignin monomer composition, increased enzymatic hydrolysis efficiency, and improved biomass conversion potential. However, similar to traditional *bmr* mutations, genome-edited lines may display trade-offs such as reduced stem strength or altered stress responses depending on the gene targeted and the extent of enzyme disruption. A summary of representative genome-editing studies targeting lignin biosynthesis genes in sorghum and related grasses is presented in **Table 9**. Significantly, precisely adjusted modifications that maintain residual enzyme activity might reduce growth disadvantages, offering a more advantageous option than conventional mutagenesis. The modification of transcription factors and regulatory elements enhances the ability to precisely alter lignin characteristics. Importantly, genome editing enables the transition from traditional lignin reduction strategies to rational lignin optimization. By combining omics-derived regulatory insights with targeted editing, it becomes possible to design sorghum genotypes with tailored lignin composition, improved biomass processability, and minimal agronomic penalties. Such precision-engineering approaches are likely to represent the next generation of lignin-improvement strategies for C_4_ bioenergy crops. These studies demonstrate that genome editing provides a flexible framework for modifying lignin biosynthesis at both structural gene and regulatory levels, enabling more precise control of lignin traits than traditional mutagenesis approaches (**Table 9**).

**Table 9:** Genome editing strategy to target lignin biosynthesis genes in sorghum and related grasses.

Genes to be edited	Editing strategy	Target pathway role	Reported phenotype	Agronomic observations
COMT (SbCOMT)	CRISPR/Cas knockout or targeted mutagenesis	Syringyl lignin biosynthesis	Reduced S-lignin content, altered S/G ratio, improved saccharification	Generally moderate growth effects; potential susceptibility to lodging depending on allele strength
CAD (SbCAD2)	CRISPR/Cas gene disruption	Final reduction step in monolignol biosynthesis	Increased aldehyde incorporation into lignin; improved digestibility	Possible reduction in stem strength; agronomic effects depend on background genotype
CCR (SbCCR)	CRISPR/Cas targeted mutagenesis	Early monolignol biosynthesis	Reduced total lignin content and enhanced sugar release	Strong knockouts may impair vascular development and biomass yield
Regulatory TFs (NAC/MYB)	Promoter editing or gene knockout	Transcriptional control of lignin pathway	Altered expression of multiple lignin biosynthesis genes	Potential to fine-tune lignin levels with fewer structural penalties

COMT; Scully et al., 2016CAD; Saballos et al., 2008 & Palmer et al., 2008CCR; Van Ackre et al., 2014NAC/MYB ; Zhao and Dixon, 2011.

**Figure 4:**
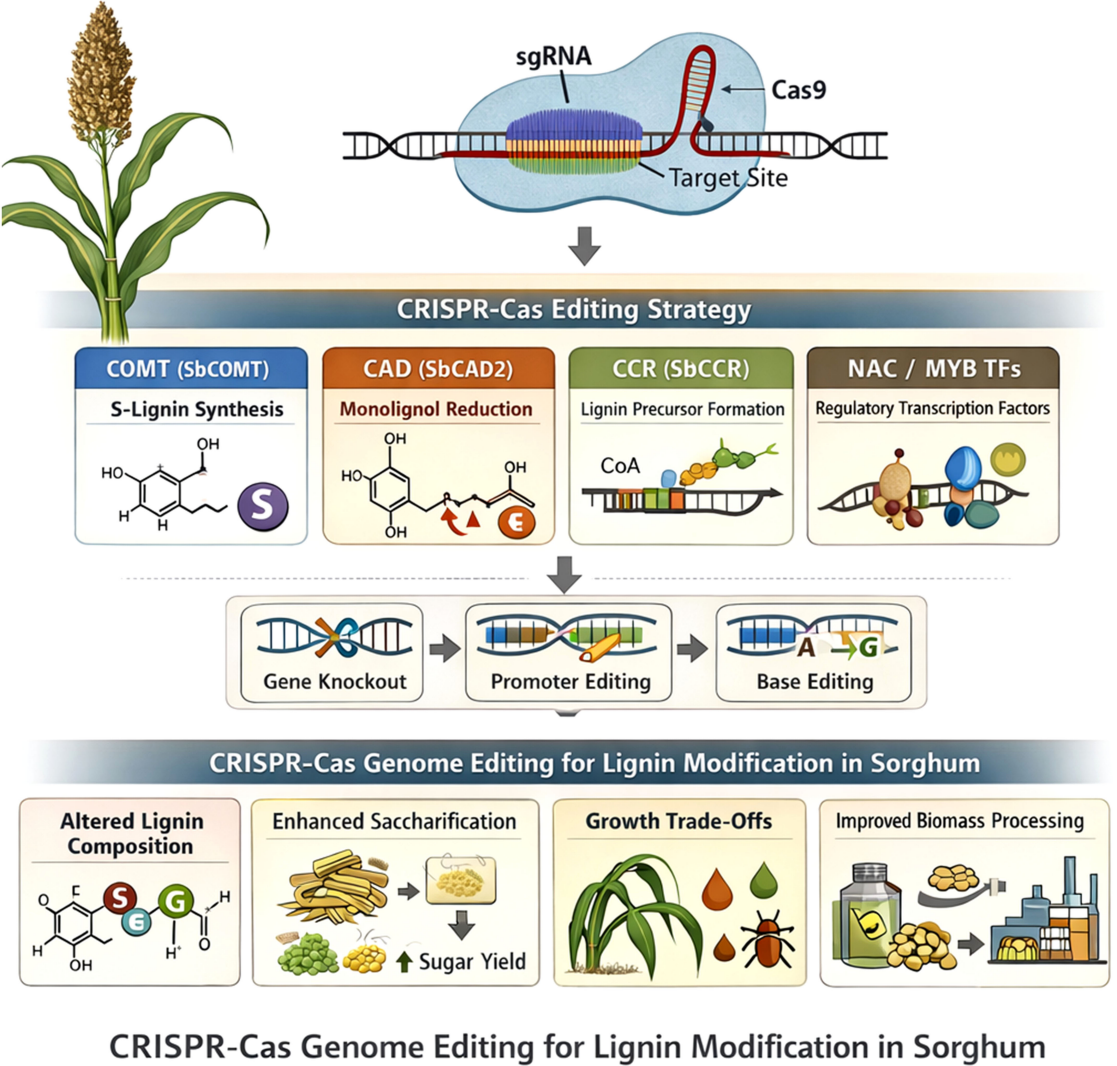
CRISPR–Cas genome editing strategies for modifying lignin biosynthesis in sorghum. This diagram illustrates how CRISPR–Cas genome editing can be used to modify key genes involved in lignin biosynthesis and regulation in *Sorghum bicolor*. Guide RNA (sgRNA) directs the Cas9 nuclease to target specific genomic sites, enabling precise gene modifications. The main target genes include COMT (caffeic acid O-methyltransferase), which plays a role in syringyl lignin formation; CAD (cinnamyl alcohol dehydrogenase), responsible for the final step in monolignol biosynthesis; CCR (cinnamoyl-CoA reductase), involved in early monolignol precursor formation; and regulatory transcription factors like NAC and MYB that control secondary cell wall biosynthesis. Genome editing methods may involve gene knockouts, promoter editing, or base editing to modify enzyme activity and lignin deposition. These changes can alter lignin composition and structure, improve enzymatic saccharification and sugar release, and enhance biomass processing efficiency for forage and bioenergy uses. However, such modifications may also cause trade-offs in agronomic traits, such as stem strength or stress response changes, underscoring the need for balanced lignin engineering approaches.

Recent advances in genome editing technologies provide unprecedented opportunities to precisely manipulate lignin biosynthesis pathways in sorghum. CRISPR/Cas systems enable targeted modification of key structural genes involved in monolignol biosynthesis, including COMT, CAD, and CCR, as well as regulatory transcription factors such as NAC and MYB proteins that control secondary cell wall formation. Unlike classical brown midrib (*bmr*) mutants generated through random mutagenesis, genome editing allows the creation of specific allelic variants that fine-tune enzyme activity or transcriptional regulation, thereby enabling lignin optimization rather than indiscriminate lignin reduction. These targeted modifications can alter lignin composition, improve biomass saccharification efficiency, and enhance suitability for bioenergy conversion while minimizing negative agronomic effects. A conceptual overview of CRISPR-based lignin engineering strategies in sorghum is illustrated in **Figure 4**.

The integration of multi-omics data enables the development of predictive models that associate genotype with phenotype in lignin-altered sorghum. These models enable rational design techniques that reconcile diminished recalcitrance with agronomic resilience. By pinpointing critical regulatory nodes and metabolic limitations, systems biology methodologies can inform the selection of target genes and editing procedures that provide favorable results (Wang et al., 2018). In sorghum, integrative analyses of genomic and transcriptomic data have already identified extensive gene networks controlling lignin biosynthesis and phenylpropanoid metabolism, highlighting numerous candidate genes that influence lignin content and composition (Niu et al., 2025).

Such systems-biology approaches help identify key regulatory nodes and metabolic bottlenecks within the lignin biosynthetic pathway, allowing researchers to prioritize target genes for crop improvement and metabolic engineering strategies (Vishnuvardhan et al., 2025).

**Figure 5:**
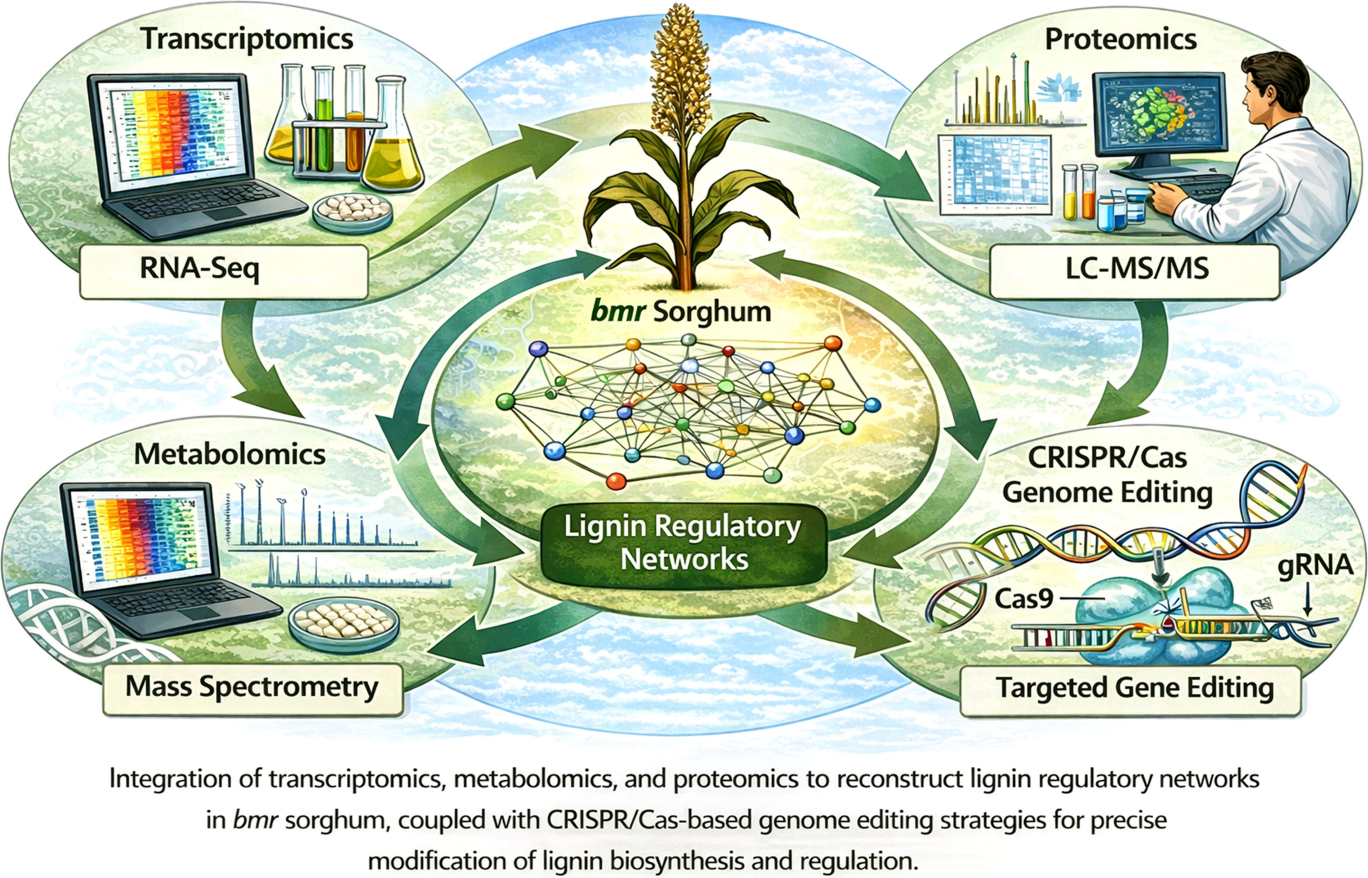
Integrative multi-omics and genome-editing framework for deciphering lignin regulatory networks in sorghum. This schematic illustrates how multiple omics approaches, including transcriptomics, proteomics, and metabolomics, can be integrated to identify key regulators of lignin biosynthesis in *Sorghum bicolor*. The framework highlights how candidate genes involved in monolignol biosynthesis and lignin polymerization can be prioritized using systems-level datasets and subsequently validated through CRISPR/Cas-based genome editing. The figure represents a conceptual workflow for lignin engineering in sorghum.

With the expansion of data resources and advancements in computational tools, the integration of omics-driven insights with genome editing is poised to expedite the creation of next-generation *bmr* sorghum varieties optimized for forage quality and renewable energy production. This integrative multi-omics framework facilitates the identification and functional validation of candidate genes involved in lignin biosynthesis and enables precise manipulation of lignin traits through genome editing approaches (**Figure 5**).

While brown midrib (bmr) sorghum lines are well-studied for enhancing forage digestibility, their significance in bioenergy has also grown (Vermerris et al., 2011; Sattler et al., 2010b). In addition to traditional bioethanol, lignin-altered sorghum biomass holds promise for various renewable energy pathways, such as lignocellulosic biofuels, thermochemical processes like pyrolysis and gasification, and the creation of bio-based chemicals and biochar (Rooney et al., 2007; Vermerris et al., 2011). Recent transcriptomic and metabolomic analyses of sorghum bmr mutants (e.g., *bmr6* and *bmr12*) have deepened understanding of lignin biosynthesis and its regulatory networks (Sattler et al., 2009; Saballos et al., 2009; Scully et al., 2016). These studies have pinpointed key genes in the phenylpropanoid pathway, including those for cinnamyl alcohol dehydrogenase (CAD) and caffeic acid O-methyltransferase (COMT), along with related transcriptional regulators affecting cell wall structure (Saballos et al., 2009; Sattler et al., 2009; Vermerris et al., 2011). However, critical questions remain about how lignin modification impacts carbon allocation, plant fitness, and biomass yield in field conditions (Scully et al., 2016; Hao et al., 2021).

### 5.4 Breeding approaches and implementation challenges for bmr sorghum

Despite significant progress in the identification and characterization of brown midrib (*bmr*) mutants, several scientific challenges remain in translating lignin modification into agronomically robust sorghum cultivars. One of the central challenges is balancing reduced lignin content with the maintenance of plant structural integrity, vascular function, and resistance to lodging and pathogens. Excessive lignin reduction can compromise stem strength and increase susceptibility to environmental stress. Therefore, future strategies must focus on optimizing lignin composition rather than simply reducing lignin concentration. Emerging approaches integrating quantitative genetics, multi-omics analyses, and genome editing provide promising solutions by enabling precise modification of key regulatory nodes in lignin biosynthesis while preserving essential physiological functions. The effective use of brown midrib (*bmr*) sorghum depends on converting molecular and physiological knowledge into superior cultivars suited for various agroecological environments. Initial *bmr* mutants served mainly as research subjects, frequently exhibiting yield reductions, susceptibility to lodging, and heightened disease susceptibility, thereby constraining their direct agricultural applicability.

The effective deployment of *bmr* alleles in superior backgrounds necessitates breeding strategies that reconcile enhanced biomass quality with strong field performance. Genomic selection methods utilizing realized relationship matrices might improve selection precision for intricate lignin-related and agronomic variables, therefore expediting the incorporation of *bmr* alleles into breeding programs (Hayes et al., 2009). Subsequent endeavors have concentrated on introgressing *bmr* alleles into high-performing genetic backgrounds to amalgamate improved biomass digestibility with satisfactory yield and stability (Saballos et al., 2008).

Traditional backcross breeding continues to be extensively employed to integrate *bmr* characteristics into adapted germplasm. This method preserves advantageous agronomic traits while implementing specific lignin alterations. The primarily recessive characteristics of *bmr* alleles require meticulous selection and comprehensive field assessment to guarantee consistent expression and uniform performance across various conditions.

### 5.5 Marker-assisted and genomic selection approaches

Progress in molecular marker technologies has markedly improved the efficacy of *bmr* breeding. The development of gene-based markers for critical *bmr* loci, including *bmr*6 (SbCAD2) and *bmr*12 (SbCOMT), has facilitated marker-assisted selection (MAS) to monitor advantageous alleles during breeding cycles. MAS diminishes dependence on phenotypic screening, which may be affected by environmental variability and developmental phases (Burow et al., 2019; Collard and Mackill, 2008). MAS has already been applied in sorghum to introgress the *bmr*6 allele into elite genetic backgrounds through marker-assisted backcrossing, improving lignin-related traits while maintaining desirable agronomic performance (Reddy et al., 2025).

Recent advancements in genomic selection methodologies utilizing genome-wide marker data have demonstrated potential for enhancing complex traits related to *bmr* sorghum, including as yield stability, lodging resistance, and stress tolerance (Madhusudhana, 2019). Genomic selection enables the concurrent enhancement of lignin characteristics and agricultural performance by accounting for the aggregate impacts of numerous loci, hence expediting cultivar development.

An innovative technique to augment the value of *bmr* sorghum involves the pyramiding of multiple *bmr* alleles or the integration of *bmr* mutations with complimentary features. For instance, combining *bmr*12 with moderate-effect alleles that affect cell wall cross-linking or transcriptional control may further diminish recalcitrance without increasing growth penalty. Pyramiding should be undertaken with caution, since excessive lignin removal may undermine structural integrity and stress resilience (Sattler et al., 2010a; Sattler et al., 2014; Reddy et al., 2024).

Allele stacking may also entail the integration of *bmr* mutations with characteristics such as diminished plant height, enhanced stem diameter, or optimized root architecture to mitigate lodging risk. These integrative tactics signify a transition from enhancing individual traits to optimizing sorghum ideotypes at a systems level (Erbetta et al., 2024).

A significant obstacle in the deployment of *bmr* sorghum is achieving uniform performance across varied conditions. Genotype-by-environment interactions markedly alter lignin deposition, growth, and stress responses, hence influencing the expression and applicability of *bmr* characteristics (Oliveira et al., 2020). Multi-location field trials are crucial for selecting genotypes with consistent performance and for comprehending how environmental conditions influence lignin-related traits (Rajesh-Kumar et al., 2025).

In areas characterized by water scarcity or thermal stress, the benefits of *bmr* sorghum may be enhanced owing to sorghum’s intrinsic stress resilience and the possibility of augmented carbon use efficiency. In high-input systems, the hazards of lodging and disease may be heightened, requiring specialized management strategies and genetic interventions.

### 5.6 Regulatory and acceptance considerations

The regulatory framework for brown midrib (*bmr*) sorghum is contingent upon the technique employed to cultivate the trait. Conventional *bmr* cultivars, obtained through natural or engineered mutations, typically encounter low regulatory restrictions and have been extensively integrated into fodder systems. Conversely, genome-edited or transgenic methods for lignin alteration may face heightened regulatory oversight, which differs by jurisdiction and policy framework. Lignin polymerization is increasingly recognized as a spatially regulated process that entails coordinated enzyme localization and the construction of cell wall microdomains (Barros et al., 2015).

Public perception and stakeholder approval are essential considerations in the implementation of *bmr* sorghum, especially for bioenergy applications. Clear communication regarding the agronomic, nutritional, and sustainability advantages, along with potential dangers, of lignin-modified crops is crucial to build trust among farmers, industry stakeholders, and regulators.

The successful implementation of *bmr* sorghum necessitates synchronization between breeding goals and biorefinery specifications. Characteristics that improve biomass processability, such as diminished lignin content and modified polymer composition, must be weighed against logistical factors like biomass yield, storage stability, and transit efficiency (Appiah-Nkansah et al., 2018). Collaboration among plant breeders, agronomists, and bioenergy engineers is essential for developing sorghum cultivars suited to certain biorefinery designs (Yang et al., 2021).

By combining molecular breeding with a comprehensive understanding of biomass consumption, *bmr* sorghum can be effectively established as a fundamental feedstock for forage-based livestock systems and renewable energy generation. While significant progress has been made in understanding and utilizing brown midrib mutations, continued advances in genomics, systems biology, and genome editing are expected to open new opportunities for optimizing lignin composition and improving biomass utilization in sorghum.

### 5.7 Future directions in lignin engineering

Initial endeavors to enhance biomass usage in sorghum mostly concentrated on diminishing total lignin content, predicated on the premise that reduced lignin will inevitably result in increased digestibility and enhanced biofuel production. Although brown midrib (*bmr*) mutants have evidently shown the advantages of lignin reduction, increasing research indicates that enhancing lignin composition and structure may be more efficacious and agronomically sustainable than indiscriminate lignin suppression (Li et al., 2010; Bonawitz and Chapple, 2013).

Future methods are consequently focusing on optimizing lignin characteristics, including altering monomer ratios, interunit connections, and polymer branching patterns, to improve processability while maintaining critical structural and protective roles. *bmr* sorghum offers a significant genetic foundation for investigating these intricate alterations and for pinpointing ideal lignin configurations suited to particular applications.

The notion of “designer lignin” has garnered attention as progress in molecular genetics and synthetic biology facilitates the precise alteration of lignin biosynthesis. Novel monomers can be introduced, enzyme specificities altered, or metabolic fluxes redirected to construct lignin polymers that are more amenable to depolymerization or selective cleavage during processing (Mottiar et al., 2016; Zhao et al., 2024). *bmr* mutants in sorghum demonstrate the viability of route reconfiguration by direct and indirect genetic modifications. Future study may concentrate on integrating *bmr*-like changes with the engineered inclusion of labile bonds or chemically different monomers, thus producing lignin structures optimal for further valorization. These methods offer potential for converting lignin from a resistant waste into a profitable renewable resource.

The economic viability of lignocellulosic biofuel systems increasingly depends on efficient lignin valorization. Lignin may be transformed from a mere low-value combustion fuel into high-value coproducts, including aromatic compounds, adhesives, resins, and advanced materials like carbon fibers (Ragauskas et al., 2014).

Integrated lignin valorization technologies that merge chemical depolymerization with biological funneling present a viable approach to transform heterogeneous lignin streams into specific, value-added products. Linger et al. (2014) shown that integrating catalytic lignin depolymerization with tailored microbial pathways facilitates the effective transformation of lignin-derived aromatic intermediates into specific compounds, thereby establishing a fundamental framework for lignin-first biorefinery concepts. Their research demonstrates that the structure of upstream lignin significantly affects the efficiency of downstream catalytic and biological conversions.

Modified lignin derived from brown midrib (*bmr*) sorghum may be especially suitable for valorization pathways owing to its changed structure and decreased condensation. These adjustments can augment depolymerization efficiency, raise product selectivity, and elevate the overall value obtained from sorghum biomass. Harmonizing in planta lignin engineering approaches with distinct valorization objectives constitutes a pivotal area in bioenergy research.

The diverse and resistant characteristics of natural lignin continue to provide a significant obstacle for biological enhancement, limiting microbial depolymerization and conversion efficiency (Beckham et al., 2016). Advancements in microbial engineering, route modularization, and lignin-first processing techniques are crucial for transforming lignin-derived aromatics into fuels and high-value compounds. Significantly, upstream alterations of lignin structure in planta can profoundly affect subsequent biological valorization results. Feedstocks exhibiting diminished lignin recalcitrance are becoming acknowledged as essential for the economically feasible synthesis of biohydrogen and other advanced biofuels from lignocellulosic biomass (Bhatia et al., 2022).

*bmr* sorghum possesses significant implications for climate-resilient agriculture beyond its industrial applications. Sorghum’s intrinsic resilience to drought, high temperatures, and suboptimal soils establishes it as a pivotal crop during climate fluctuations. Modification of lignin may augment resilience by enhancing carbon utilization efficiency and enabling more adaptable resource allocation under stress situations (Abreha et al., 2022; Romana et al., 2017).

Future study should investigate the interplay between lignin modification and stress tolerance mechanisms to guarantee that *bmr* sorghum varieties retain resilience under fluctuating environmental conditions. Incorporating lignin engineering with characteristics like enhanced water-use efficiency and nutrient absorption will be crucial for optimizing the sustainability advantages of *bmr* sorghum. Early efforts in lignin engineering primarily focused on reducing total lignin content to improve biomass digestibility and biofuel conversion. However, increasing evidence indicates that the optimization of lignin composition and polymer structure may provide more sustainable solutions than indiscriminate lignin reduction. This shift reflects a broader transition in plant biotechnology—from single-gene manipulation toward systems-level approaches that integrate genetics, metabolic regulation, and crop performance.

To fully realize the potential of *bmr* sorghum, it is essential to integrate fundamental research, breeding, agronomy, and industrial processing closely. Interdisciplinary partnerships will be essential for synchronizing lignin modification efforts with practical limitations and opportunities throughout the value chain. Improvements in phenotyping, modeling, and data integration can expedite the conversion of laboratory findings into practical applications (Mullet et al., 2014; Singh et al., 2022).

**Figure 6:**
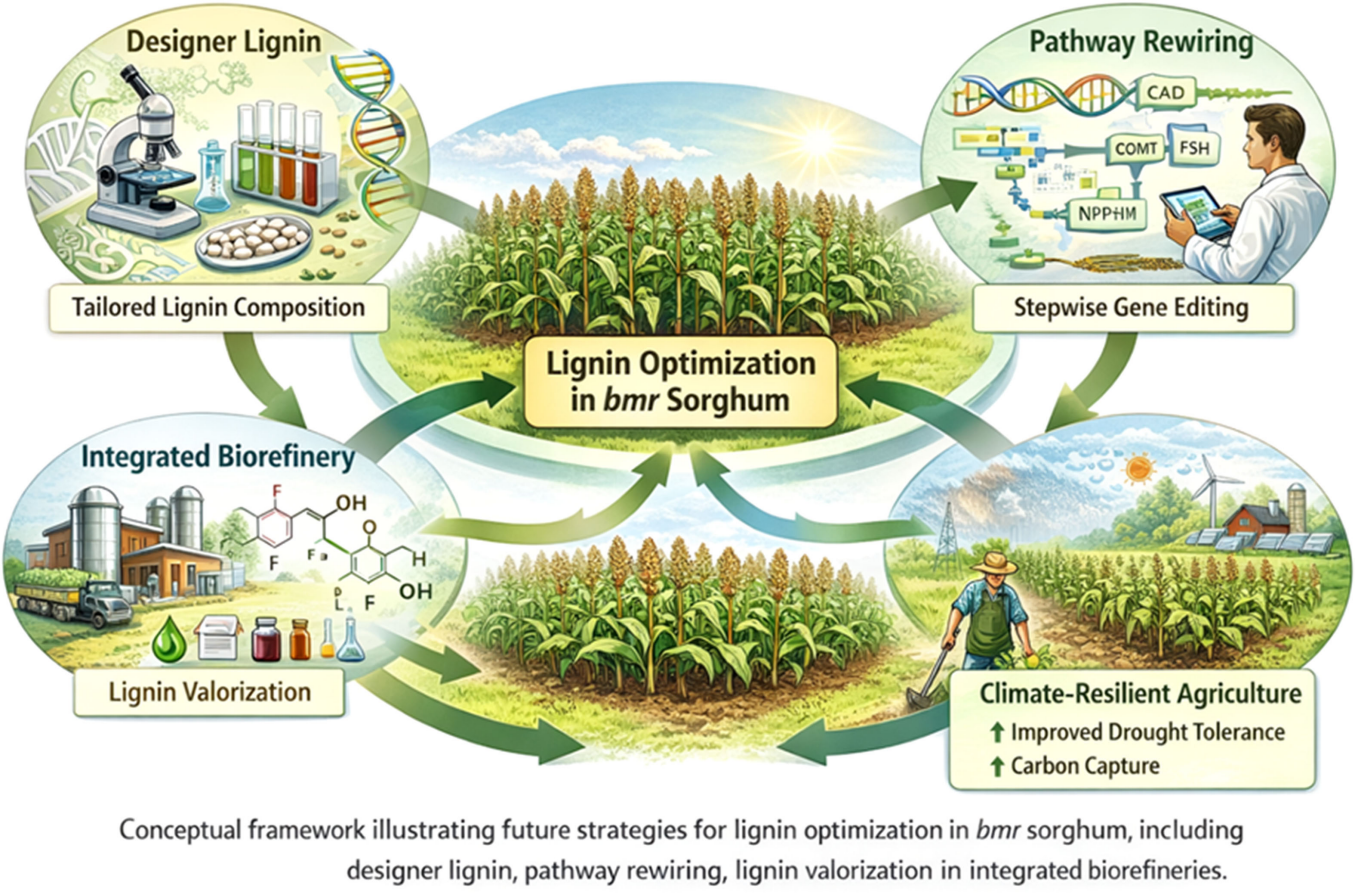
A conceptual framework for the optimization of lignin in *bmr* sorghum. It underscores strategies aimed at modifying lignin quantity and quality to enhance biomass utilization and strengthen climate resilience. Using gene-editing techniques, specific lignin traits can be achieved. In this process, precise editing of pivotal monolignol biosynthesis genes, such as CAD, COMT, and F5H, was performed. Lignin properties facilitate more efficient biorefinery processes by enabling its conversion into valuable chemicals, bioethanol, and biomaterials, thereby supporting climate-resilient agriculture.

With ongoing research, *bmr* sorghum is set to function both as a target for crop enhancement and as a model system for elucidating and manipulating lignification in C_4_ grasses. Insights gained from sorghum are expected to enhance lignin engineering initiatives across additional bioenergy crops, thereby magnifying the overall significance of this research (**Figure 6**).

## 6 Conclusion

Brown midrib (*bmr*) sorghum represents one of the most important genetic resources for improving the utilization of lignocellulosic biomass in both forage and bioenergy systems. Research on *bmr* mutants has provided critical insights into lignin biosynthesis, its genetic regulation, and its impact on cell wall structure and biomass recalcitrance. These discoveries have enabled the development of sorghum genotypes with improved fiber digestibility for livestock production and enhanced saccharification efficiency for biofuel conversion. Collectively, research on sorghum brown midrib mutants has transformed our understanding of lignin biosynthesis and its implications for agriculture and renewable energy systems. Brown midrib (*bmr*) mutants in *Sorghum bicolor* have significant role in advancing our understanding of lignin biosynthesis, its regulation, and functional significance in C_4_ grasses. By linking observable phenotypes *bmr*to distinct genetic and biochemical changes, *bmr* sorghum provides a reliable framework for analyzing the phenylpropanoid pathway and investigating the interactions among lignin composition, cell wall structure, and plant efficacy. Lignin serves multiple biological functions beyond providing structural support, underscoring the necessity for balanced engineering approaches in bioenergy crops (Liu et al., 2018; Sattler and Funnell-Harris, 2013).

Investigations of key *bmr* loci, namely *bmr*2, *bmr*6, *bmr*12, *bmr*19, and *bmr*30, indicate that targeted lignin alteration might significantly reduce biomass recalcitrance while maintaining functional secondary cell walls. The data indicate that lignin quality, rather than mere amount, is a crucial factor influencing fodder digestibility and bioenergy conversion efficiency. The varied phenotypic results of several *bmr* alleles highlight the significance of route location, metabolic integration, and regulatory compensation in influencing lignin characteristics. Furthermore, lignin alteration might affect plant–pathogen interactions through modified phenolic profiles, highlighting the importance of integrative breeding techniques (Funnell-Harris et al., 2017).

The enhanced biomass productivity and resource-use efficiency of C_4_ grasses render them ideal for cellulosic biofuel production, especially when integrated with lignin-modifying characteristics (van der Weijde et al., 2013). From a practical standpoint, *bmr* sorghum improves fiber digestibility, voluntary feed consumption, and animal performance in forage systems, while decreasing pretreatment intensity, enhancing saccharification efficiency, and augmenting bioethanol production in bioenergy applications. Despite ongoing concerns about lodging susceptibility and disease sensitivity, these issues can be mitigated through intelligent breeding, optimal management, and meticulous selection of genetic backgrounds. Meta-analytic analysis demonstrates that *bmr* sorghum consistently enhances fodder digestibility and nutrient utilization relative to standard cultivars (Widodo et al., 2025).

The convergence of systems biology, transcriptomics, and genome editing technologies marks a new era in lignin research, enabling precise and systematic manipulation of lignin biosynthesis and regulation. These technologies facilitate the creation of sorghum varieties with enhanced lignin characteristics designed for applications, such as improved biofuels and lignin-derived coproducts. In the future, lignin engineering will likely transition from basic reduction to advanced optimization and valorization tactics that improve economic and environmental sustainability.

In summary, brown midrib sorghum epitomizes the intersection of essential plant biology, agricultural enhancement, and renewable energy investigation. Utilizing genetic variety, contemporary biotechnological instruments, and interdisciplinary cooperation, *bmr* sorghum can enhance climate-resilient agriculture and sustainable bio-based businesses. Looking forward, the application prospects of *bmr* sorghum extend beyond traditional forage systems. The integration of *bmr* alleles into high-yielding sorghum germplasm offers opportunities to develop dual-purpose cultivars suitable for both livestock feed and lignocellulosic bioenergy production. Advances in genome editing, systems biology, and precision breeding are expected to facilitate the development of sorghum varieties with optimized lignin composition that maintain agronomic stability while improving biomass conversion efficiency. Such innovations will strengthen the role of sorghum as a climate-resilient crop capable of supporting sustainable agriculture, renewable energy generation, and integrated biorefinery systems. Ongoing investment in this research domain not only assures progress in sorghum enhancement but also yields widely relevant insights into lignin engineering among grass species.
